# Modeling of methane emissions from waste disposal sites at selected Egyptian governorates and potential energy production from waste-to-energy projects

**DOI:** 10.1038/s41598-024-73572-9

**Published:** 2024-10-23

**Authors:** Shady Mohamed Naguib Mohamed Khafagy, Amr El Sammak, Karim Emara

**Affiliations:** 1 Faculty of Science, Alexandria University, Alexandria, Egypt; 2 Faculty of Engineering Mataria, Helwan University, Cairo, Egypt

**Keywords:** Municipal solid waste, Waste-to-energy, Methane, Greenhouse gases, Potential energy, Incineration, Landfill gas to energy (LFGE), Economic feasibility, Energy, Biogas, Climate-change mitigation

## Abstract

Waste and energy sectors have significant contributions to the greenhouse gas (GHG) emissions caused primarily by the population expansion. Waste-to-Energy (WtE) is introduced to address the issue raised by both sectors simultaneously through utilization of the potential energy stored in municipal solid waste (MSW) as well as offsetting GHG emissions. Limited research have been conducted in Egypt to assess the current situation of MSW management and associated methane emissions. The current study focused on estimating the baseline methane emissions for six Egyptian governorates and determining the energy production potential from WtE projects. To achieve this aim, three scenarios have been assessed: Baseline, Landfill Gas to Energy (LFGE), and Incineration scenarios. Key results revealed that a total of 3.7 million tonnes of methane would be emitted from all studied governorates generated over 50 years. Incineration also found to be more favorable in all governorates in terms of energy production, quantity of avoided GHG emissions, and in terms of economic viability over LFGE. Implementing incineration in all governorates would generate about 5.6 TWh energy annually and could avoid about 5 Mt CO_2_ eq annually in comparison to LFGE that would generate about 0.6 TWh annually and could avoid about 2.5 Mt CO_2_ eq annually. In terms of economic viability of WtE projects, while they were generally not economically viable under the assumptions made in the current study, incineration technology deemed promising, but policy adjustments, such as competitive Feed-in Tariff (FiT) rates and the inclusion of gate fees, are necessary. Specific minimum gate fees and FiT were identified for each governorate, providing essential guidance for decision makers to ensure the viability of WtE implementation. This study would support the decision makers in assessing technically and financially feasible options for WtE technologies in the selected governorates.

## Introduction

The human race is currently facing the inevitable consequences of climate change and global warming that is typically associated with the increase in greenhouse gas (GHG) emissions^1^. The GHG levels reached 52.4 Gt CO_2_ eq in 2019, where the contribution of GHG gases were carbon dioxide (CO_2_) with 72%, methane (CH_4_) with 19%, and nitrous oxide (N_2_O) with 6% of the total GHG levels^2^. According to^3^, GHG emissions from the waste sector represent the third highest GHG contributor with about 3% of global emissions. Significant methane emissions are emitted to the atmosphere from the waste sector, especially from poorly managed municipal solid waste (MSW) disposal sites and unmanaged landfills as a result of the decaying processes of MSW^2,4,5^. Landfill methane emissions contribute to about 12% of global methane emissions^2^. Methane emission is substantial as it has a global warming potential (GWP) of more than 28 times that of carbon dioxide^6^. In Egypt, the waste sector generated considerable GHG emissions in 2015 that amounted to 26.4 million tonnes CO_2_ eq., which represent about 8.1% of national GHG emissions^7^.

One of the main drives for the increasing trend in methane emissions from the waste sector is the unprecedented surge in the global MSW generation as a direct result of population growth. As it anticipated that the global MSW quantities would reach 9.5 billion tonnes in 2050^8^, while Egypt is considered one the highest waste generation countries in the African continent, with a generation rate of about 29 million tonnes of MSW annually^9^. The main MSW management methods around the world are landfilling, composting and incineration^10^, where the landfilling method has the major share of 37% of the total MSW, followed by open dumping, composting, and incineration with shares of 33%, 18%, and 11%, respectively^11^. In Egypt, around 90% of the generated MSW is directed to open dumpsites and unmanaged landfills^12^.

Synchronically, the growing energy demand due to the population expansion globally has always stressed the need for consuming nonrenewable fossil fuels such as coal, oil and natural gas in the production of electricity^8^. In 2021, Egypt has generated 204.8 TWh of electricity to cover local energy demand^13^. Energy sector also has a pivotal contribution to the total anthropogenic GHG emissions with more than 90%^14^. The combined problem of the increasing energy demand and the improper management of MSW has created the need for alternative renewable energy sources that simultaneously help in reducing MSW quantities as well as offsetting GHG emissions^14,15^. Waste-to-Energy (WtE) is the key technology for harnessing potential energy content stored in the organic fraction of the MSW and transferring it into electricity^8,16,17^. It should be pointed out that potential energy produced from MSW through WtE is mainly relying on waste characterization that varies depending on the level of urbanization, population, and income of different geographical locations^18^. As the WtE projects have the ability to produce electricity from MSW utilization, they could offset the fossil carbon emissions from typical power stations producing the same amount of energy produced from the WtE projects^10^.

Biochemical and thermochemical conversion are the main processes in WtE technologies^8^. Biochemical conversion encompasses the breakdown of biodegradable organic fractions of the MSW in the presence of bacteria^8,17^. While thermochemical conversion entails the breakdown of the fossil carbon in the organic fractions of the MSW under the effect of high temperature^8^. The main WtE technologies under the biochemical and thermochemical conversion processes are landfill gas energy (LFGE) and incineration, respectively^16^. Various commercially available WtE technologies are currently available include incineration, landfill gas recovery systems, gasification, anaerobic digestion, pyrolysis, bioethanol production^11^. Selecting the most suitable WtE technology for a specific region involves considering a range of factors, encompassing technical, economic, environmental, and social aspects. These criteria include assessing the technology’s maturity level, waste composition and properties, land area requirements, capital and maintenance costs, technological complexity, and overall efficiency^8^. Table 1 provides a comparison of the two primary WtE technologies based on these factors, as obtained from^8,19,20^. Nevertheless, variety of the available WtE technologies, the current study focused specifically on LFGE and incineration, as per the Egyptian Prime Minister’s decree number 41/2019 issued in 2019^21^. This decree promotes these projects through a Feed-in-Tariff (FiT) scheme without mentioning other technology types. Additionally, as indicated in Table 1, a crucial criterion for considering LFGE and incineration under Egyptian FIT regulations is their ability to handle mixed wastes without requiring separation from the source or pre-treatment, which aligns well with the conditions in Egypt.

**Table 1:** Technical, economic, environmental and social aspects of WtE technologies^8,19,20^.

Aspect	Parameter	Incineration	LFGE
Technical aspects	Technology advancement	Extremely high	Very high
	Received waste type	Mixed wastes	Mixed wastes
	Land requirement	Small area required	Very large area required
	Overall system efficiency	50–60%	10%
	Pre-treatment	Not required	Not required
	Volume reduction	80% to 90%	Low
	Energy potential	Extremely high	High
Economic aspects	Capital cost	155–250 USD/tonne (in developing countries) 620–700 USD/tonne (in developed countries)	10 USD/tonne (in developing countries) 155–200 USD/tonne (in developed countries)
	O&M costs	85 USD/tonne (in developing countries) 62–70 USD/tonne (in developed countries)	0.2–0.3 USD/tonne (in developing countries) 11–14 USD/tonne (in developed countries)
Environmental aspects	Potential for GHG emissions avoidance	Extremely high	High
	Dioxin and furan emissions	Very high	Extremely low
Social aspects	Social opposition	Extremely high	Less

In landfills, treatments fall into three categories: aerobic, semi-aerobic, and anaerobic^22^. Landfill gas (LFG) primarily results from the bacterial breakdown of organic fractions within MSW. The anaerobic digestion of MSW’s biodegradable organic fractions leads to the formation of landfill gas (LFG) that mainly contains methane (CH_4_) (50–60%) and carbon dioxide (CO_2_) (40–50%)^17,23^. The process of LFG generation involves three phases: bacterial decomposition, chemical interactions, and volatilization. The generated LFG undergoes treatment to remove hydrogen sulfides through scrubbers to ensure that the treated LFG does not corrode the metallic components of the subsequent energy production system^22^. Several conditions affect the quantities of LFG including waste characterization, quantity, climatic conditions as well as the percentage of biodegradable fractions in MSW^24^. The generated landfill gas could be utilized in LFGE technology by collecting and combusting LFG in an internal combustion engine (ICE) to produce electricity and CO_2_ through a collection of pipes and wells, which can be either active or passive. Vertical wells and horizontal trenches are employed, utilizing natural pressure gradients or pumps^8,25^. Methane emission from LFG is the only accounted for as GHG as it contributes to global warming^26^. However, neither the CO_2_ emitted from the anaerobic digestion of MSW in landfill nor that emitted as a result of combusting LFG in ICE are considered GHG as they are of biogenic origin and have no global warming potential (GWP)^27,28^. LFGE technology have several advantages including the utilization of LFG in energy production, LFGE is comparatively cheap technology and the operation of LFGE does not require skilled workers. In contrast, several bottlenecks are encountered in LFGE, such as that landfills require a large land footprint, and the leachate generated from landfill could potentially contaminate groundwater as well as the potential explosion risk from methane accumulation in the landfills^8,17^.

Incineration is a well-established thermal treatment method which stands as the most widely adopted technology for waste management globally. Incineration technologies can be categorized into three primary types: moving or fixed grate, rotary kiln, and fluidized bed combustors. The choice of incinerator depends on the characteristics and composition of the waste. For instance, waste with higher moisture content is more effectively incinerated in fluidized bed combustors, while less moist waste is typically handled using grate incinerators^8^. Among various incineration technologies, moving grate incineration stands out as a highly effective and widely commercialized option for handling non-recyclable waste^11^. In terms of land requirement, a standard incineration plant handling 300 tonnes per day typically requires around 0.8 hectares of land^19^. Incineration directly combusts the MSW in a temperature range from 800 to 1000 °C in which most of hazardous materials in the waste are destructed^8^, and the hot flue gases are cooled in water boilers to produce steam which in turn drives the steam turbine for electricity generation^8,29^. In incinerator, when organic factions of the MSW reach the necessary ignition temperature and come into contact with oxygen, they will burn. This actual combustion process occurs in the gaseous state within fractions of seconds, simultaneously releasing energy. When there is an adequate supply of oxygen and the waste has sufficient calorific value, a thermal chain reaction can occur, leading to self-sustaining combustion^22^.The produced energy from incineration is mainly dependent on the amount of fossil carbon in the MSW^30^. In order to generate viable energy from incineration, lower calorific value (LCV) of the MSW should ranges from 6000 to 7000 kJ/kg^15,31^ and the quantities of MSW should not be lower than 100 tonnes per day^32^. According to^18^, MSW incineration technology generates energy 2.8 times more than LFGE for the same amount of MSW received by both technologies. Comparing to LFGE, MSW incineration is more advantageous as it could reduce 70–80% of MSW weight and 80–90% of MSW volume^8^ as well as it has higher ratio of energy recovery^20^. Though, one of the main drawbacks of incineration technology is that MSW combustion process emits carcinogenic dioxins and furans that require strict emission control and treatment measures^11,17^. Another common bottleneck of incineration technology, particularly in developing countries, is the lack of waste sorting. MSW in these regions tends to be unsorted and contains a high organic fraction with elevated moisture content. This combination can result in various operational issues, including corrosion, process unreliability, and increased auxiliary fuel consumption^11^.

With the potential of WtE projects to reduce GHG emissions comparing to the baseline status of improper MSW management, carbon emission trading mechanisms such as Clean Development Mechanism (CDM) could be a key to foster the development of such projects^33,34^. In response, Egypt has recently issued a decree number 4774 in December 2022 by the Egyptian Cabinet of Ministers (ECM) establishing carbon trading market in the Egyptian stock exchange for the exchange of Certified Emission Reductions (CERs) for carbon emission reduction projects^35^. Moreover, the Prime Minister decree number 41/2019 set a purchase price of 1.4 EGP for each kWh of electrical energy produced from WtE projects, specifically, LFGE and incineration, via Feed-in-Tariff (FiT) scheme^21^. On the other hand, the establishment of WtE projects in Egypt faces numerous obstacles that can impede their progress. The primary issue is that the FiT rate designated for WtE projects does not yield adequate income, and the current regulations do not support the collection of a gate fee, which is a charge that a WtE facility would typically receive for accepting one tonne of waste^36^.

Lately, the global increasing attention concerning climate change issues and particularly those related to waste and energy sectors has motivated researchers around the world to study the combined problem of MSW management and energy generation and their economic feasibility. Most of the researchers indicated that incineration is more favorable option than LFGE in terms of the energy produced and the potential to offset more GHG emissions^3,10,15,37^ and also in terms of economic feasibility as reported by several studies^6,8,38–40^. Table 2 includes summary of results obtained by several authors regarding landfills’ methane emissions, potential energy production from LFGE and incineration technologies in different geographic regions.

**Table 2:** summary of literature results regarding landfills’ methane emissions, potential energy from LFGE and incineration.

WtE technology	Study area	Timescale	Findings	Reference
LFGE	Shanghai, China	2005–2015	Methane quantities from Laogang Landfill in Shanghai were estimated to reach a total 0.83 Mt in 2015 from a total MSW amount of 41.8 Mt (0.02 t CH_4_/t MSW)	^5^
LFGE	Mauritius	1997–2018	Methane generation potential from Mare Chicose landfill in Mauritius was estimated to be 0.09 t CH_4_/t MSW Potential electricity generation was about 0.05 TWh (about 0.12 kWh/kg MSW) that would avoid about 0.04 Mt CO_2_ equivalent (about 0.09 t CO_2_ e/t MSW)	^61^
LFGE and Incineration	Major cities in Brazil	2011	Methane quantities from major cities in Brazil were estimated to reach 935.3 Mm^3^ (equivalent to 0.67 Mt) from a total MSW amount of 18.9 Mt (0.03 t CH_4_/t MSW) LFGE was estimated to generated about 2.3 TWh (about 0.12 kWh/kg MSW) annually from all studied cities, while incineration was estimated to generated about 8.6 TWh (about 0.45 kWh/kg MSW) annually	^15^
LFGE and Incineration	Malaysia	2000–2030	Methane generation potential from Malaysia landfills was estimated to be 0.053 t CH_4_/t MSW LFGE was estimated to generated about 0.37 kWh/kg MSW, while incineration was estimated to generated about 0.49 kWh/kg MSW	^16^
LFGE and Incineration	Kafr El-Sheikh, Egypt	2019	Methane quantities from landfills in Kafr El-Sheikh governorate in Egypt were estimated to produce an annual amount of 0.04 t CH_4_/t MSW Total potential energy from LFGE was estimated to be 0.16 TWh annually from total MSW amount of 0.92 Mt, which corresponding to 0.17 kWh/kg MSW Incineration of the same amount of waste estimated to produce 0.54 TWh of electricity annually, which corresponding to 0.56 kWh/ kg MSW	^41^

In Egypt, limited research have been conducted regarding the methane emissions from open landfills/dumpsites and the potential energy generation from MSW as could be found in the works conducted by^12,28,41–43^ and also regarding the economic feasibility of WtE technologies as could be found in the work made by^36^. A considerable effort was made by researchers to assess the current situation of MSW management in Egypt. However, several gaps have been identified, as follows: (i) studies considered static MSW amount driven from census data without taking into consideration the effect of population growth and economic status of the studied areas; (ii) there is no estimation of baseline methane emissions from the open dumpsites/unmanaged landfills; and (iii) energy estimations and cost analyses considered limited study areas without reflecting different geographic areas as well as population sizes. In that regard, the current study aims to enrich the existing literature and to support decision makers via the following objectives: (i) estimation of MSW amount for six Egyptian governorates reflecting population growth rate and gross domestic product (GDP); (ii) estimation of baseline methane emissions reflecting the current situation in the studied Egyptian governorates; (iii) determination of energy production potentials from WtE projects (LFGE and incineration); (iv) estimation of the avoided GHG emissions from the proposed WtE projects that could be transferred into CER certificates representing a possible revenue stream to such projects; and (v) assessment of economic feasibility of the WtE projects to identify which WtE technology is most suitable for each governorate considering the current FiT regulations and to assess the sensitivity of varying the different economic, technical and policy aspects on the WtE projects’ profitability.

## Methods

**Fig. 1:**
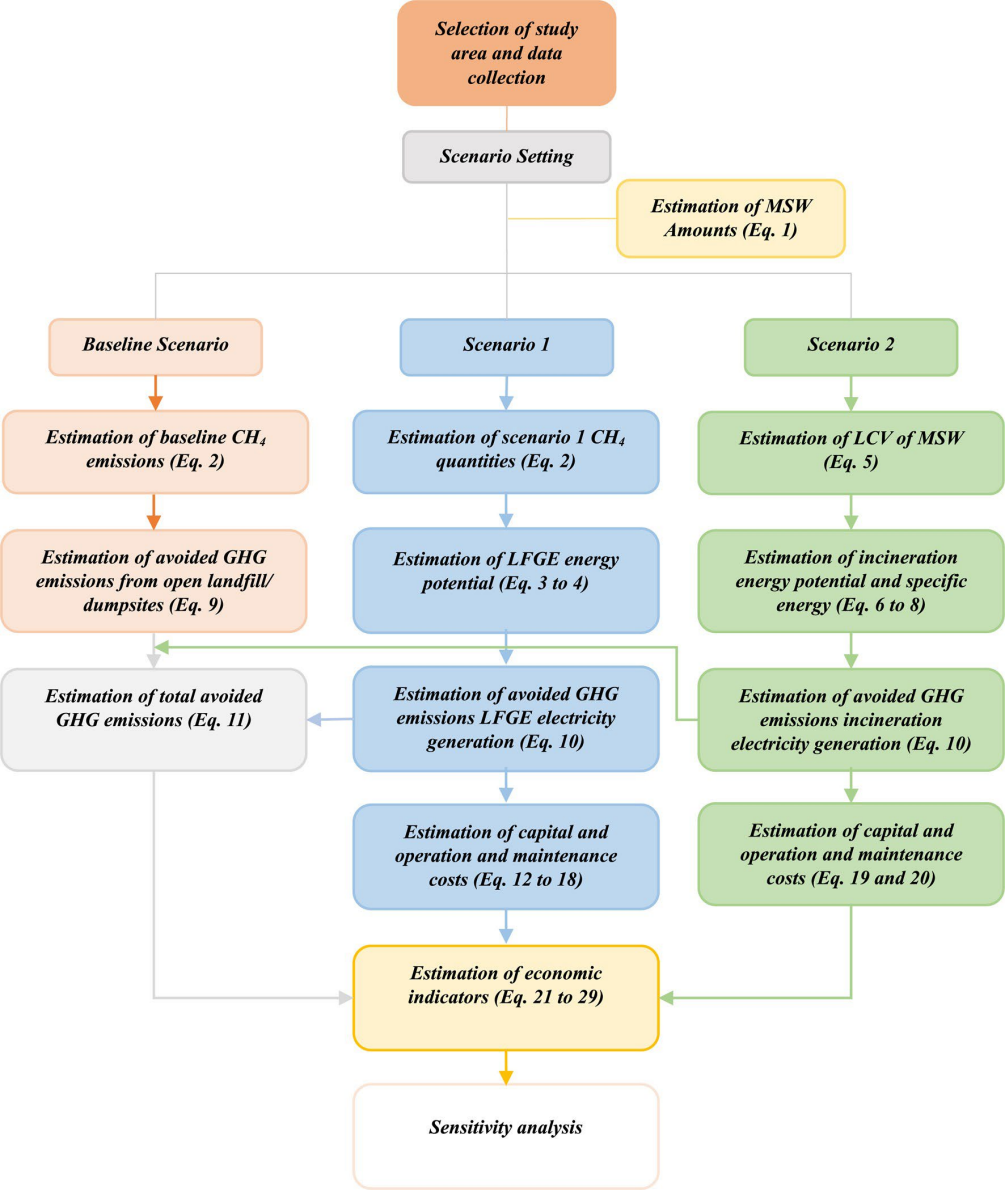
Research methodology flow diagram.

The research methodology adopted in the current study is depicted in Fig. 1. The study adopted a staged approach, where in the beginning data regarding population, MSW characteristics and generation rate have been acquired. Subsequently, scenarios under study have been identified to determine the boundaries of the study. Baseline methane emissions from open dumpsites/unmanaged landfills were estimated in the baseline scenario using Eq. 2. While, in scenario 1, methane quantities from well-managed landfill have been estimated using Eq. 2, followed by potential energy calculations through Eqs. 3 and 4. In scenario 2, the lower calorific value (LCV) of MSW has been calculated by Eq. 5, followed by potential energy calculations through Eqs. 6–8. The total avoided GHG emissions from the displacement of open dumpsites and that from electricity generation of WtE technology were estimated using Eqs. 9–11. The economic feasibility of the proposed two WtE technologies have been assessed using Eqs. 12–29. Eventually, sensitivity analysis was conducted for both WtE technologies to assess the impact of possible variations in economic, technical, and policy aspects on the projects’ profitability.

Nevertheless, the importance of Life Cycle Assessment (LCA) as a comprehensive methodology for evaluating environmental impacts across the entire life cycle of any process, the adopted methodology focused on the investigation of methane emissions, generated electricity, avoided GHG emissions and economic feasibility of the key stage of the life cycle of the waste management, which is the waste processing in WtE technologies (LFGE and incineration). An assumption was made that other lifecycle stages, such as waste transportation, were insignificant due to the equal distance between WtE facilities. Consequently, any changes in transportation resulting from waste redirection were considered insignificant.

### Study area and data collection

**Fig. 2:**
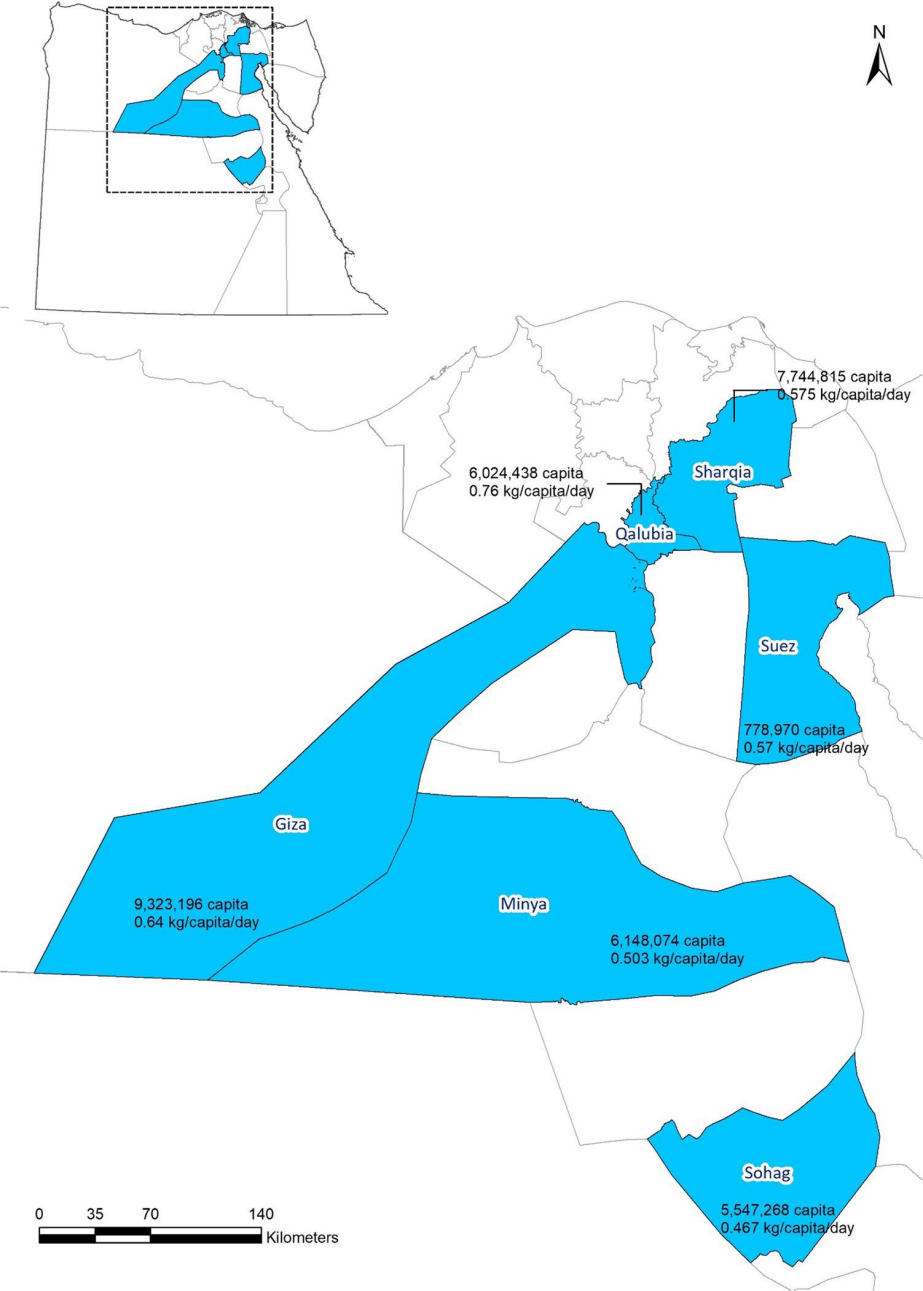
Study area showing the selected governorates, population and waste generation rate, created by Esri, ArcGISOnlineGeoEye, https://www.arcgis.com/home/webmap/viewer.html.

Egypt is divided geographically into 27 governorates^42^. In the current study, six governorates have been selected reflecting the geographical distribution as well as the different economic and population features, as shown in Fig. 2. Population of each governorate in the year 2022 has been acquired from Central Agency for Public Mobilization and Statistics (CAPMAS) in Egypt^44^, while waste generation rates and waste characteristics have been acquired from MSW masterplan developed for each governorate maintained at the Waste Management Regulatory Authority (WMRA), as provided in Table 3. Moreover, annual electricity consumption of each of the studied governorate has been estimated based on the per capita consumption figure published by World Bank, which was 1,592 kWh/capita^69^, as shown also in Table 3.

**Table 3:** Population, annual electricity consumption, waste characteristics and waste generation rate at the studied governorates.

Governorate	Population ${P}_{c}$^(1)^	Annual electricity consumption TWh	Waste generation rate ${W}_{c}$	Paper	Garden	Organic	Textiles	Wood	Plastics	Glass	Metals	Other
Giza	9,323,196	14.8	0.64^(2)^	1.61%	0.00%	63.29%	0.35%	0.08%	14.89%	1.34%	0.24%	18.20%
Minya	6,148,074	9.8	0.51^(4)^	3.00%	2.00%	80.00%	0.00%	0.00%	7.00%	1.00%	0.00%	7.00%
Qalubia	6,024,438	9.6	0.76^(5)^	13.00%	0.00%	60.00%	3.00%	0.00%	15.00%	4.00%	5.00%	0.00%
Sharqia	7,744,815	12.3	0.57^(3)^	4.90%	0.00%	54.14%	3.58%	0.55%	19.54%	1.61%	0.63%	15.05%
Sohag	5,547,268	8.8	0.46^(6)^	11.00%	7.00%	46.00%	1.00%	1.00%	18.00%	1.00%	1.00%	14.00%
Suez	778,970	1.2	0.57^(7)^	5.28%	0.00%	53.12%	1.73%	0.23%	16.79%	0.95%	0.52%	21.38%

^(1)^^44^^(2)^^62^^(3)^^63^^(4)^^64^^(5)^^65^^(6)^^66^^(7)^^67^.

### Scenarios setting

To achieve the objectives of the current study, three scenarios have been studied, as follows:

- *Scenario 0 (Baseline)*: this scenario represents the current situation of the municipal waste management (MSW) practices conducted at each governorate which is mainly 90% of MSW is transported to open dumpsites/unmanaged landfills without energy recovery, as indicated by^12^.
- *Scenario 1*: in this scenario all MSW originally directed to open dumpsites (90%) are transported to a well-designed engineered landfills with capability of landfill gas (LFG) recovery to generate electricity through internal combustion engine (ICE).
- *Scenario 2*: in this scenario all MSW originally directed to open dumpsites (90%) are transported to incineration facilities for energy recovery.

### Estimation of MSW amount

The generation potential of municipal solid waste (MSW) is predominantly influenced by the population growth rate and the economic status of the study area. Accordingly, to estimate the MSW quantities generated at each of the studied governorate, Eq. 1, as obtained from^38^, has been used taking into consideration the gross domestic product (GDP) and population growth rate.

1 $${W}_{q}=\frac{{P}_{c} . {(1+{P}_{gr})}^{t}. {W}_{c} . {(1+{GDP}_{r})}^{t}. {T}_{a} }{1000}$$

where ${W}_{q}$ is the quantity of wastes generated from the studied governorate per annum (tonnes/year), ${P}_{c}$ is the current population of each governorate (person) (provided in Table 3), ${P}_{gr}$ is the annual population growth rate of Egypt (%), which is 1.7%^45^, $t$ is the time increment (which is equal to the subtraction of subsequent year from calculation year) (year), ${W}_{c}$ is current waste generation rate per capita of each governorate (kg/person/day) (provided in in Table 3), ${GDP}_{r}$ the annual GDP growth rate of Egypt (%), which is 3.3%^46^, ${T}_{a}$ is the number of days per annum (365 days/year) and $1000$ is factor to convert kg to tonnes.

### Estimation of baseline (Scenario 0) methane emissions

In the baseline scenario, 90% MSW quantities generated at each governorate are sent to unmanaged landfill or open dumpsites without energy recovery. As a result of waste accumulation over years in open dumpsites, the decomposition of biodegradable fractions of wastes occurs anaerobically producing mainly methane (CH_4_) and carbon dioxide (CO_2_).

To estimate methane emissions, the CDM methodological tool of estimation of emissions from solid waste disposal sites has been used, as per the equation used by^14^, shown in Eq. 2 below. The CDM methodological tool is used in the current study as in case that carbon exchange market is activated in Egypt, estimation of methane emissions should be conducted using this tool for CDM projects^47^ and also it provides reliable and precise estimations of the methane quantities at the country scale^48^.

2 $${Q}_{{CH}_{4}}= \varphi \left(1-OX\right)\frac{16}{12}F.{DOC}_{F}.MCF.\sum_{x=1}^{y}\sum_{j}{W}_{j.x}.{DOC}_{j}.{e}^{-{k}_{j}.\left(y-x\right)}.(1-{e}^{-{k}_{j}})$$

where ${Q}_{{CH}_{4}}$ is the annual methane quantity (tonnes), $\varphi$ is the model of correction factor, $OX$ is the oxidation factor reflecting the amount of methane that is oxidized in the soil or other material covering the waste, $\frac{16}{12}$ is the carbon conversion factor to methane, $F$ is the methane fraction in the LFG, ${DOC}_{F}$ is the decomposable fraction of degradable organic carbon in wastes, $MCF$ is the methane correction factor, $x$ is the starting year for waste disposal, $y$ is the final modeling year, $j$ is the category of waste type, ${W}_{j.x}$ is the amount of waste category j in the year $x$ (tonnes), ${DOC}_{j}$ is the decomposable fraction of degradable organic carbon in the waste category $j$, and ${k}_{j}$ is the decay rate constant of waste category $j$.

The current study accounts for the total waste quantity that the landfill could receive, which is identified as waste acceptance limit, which is 20 years^27,38,49^. As LFG emissions could extend beyond waste acceptance limit^50^, 50 years of modeling of methane emissions have been accounted for in the current study starting from the year 2022 ending in 2072.

Default values recommended by both the CDM Executive Board in their “*methodological tool to determine methane emissions avoided from disposal of waste at a solid waste disposal site*”^51^ as well as “*2006 IPCC Guidelines for National Greenhouse Gas Inventories*”^52^ have been used in the estimation of methane emissions from the open dumping of wastes in the six governorates, as provided in Table 4.

**Table 4:** Default values used in the CDM methodological tool.

Parameter	Default value	Note	References
$\varphi$	0.9	Accounting for model uncertainties	^51^
$OX$	0	Reflecting the current situation of open dumping in all governorates as they are not covered by any covering materials	^51^
$F$	0.5	Reflecting typical percentage of organic carbon that could be degraded	^51^
${DOC}_{F}$	0.5		^51^
$MCF$	0.6	Methane correction factor for uncategorized solid waste disposal sites	^52^
${DOC}_{j}$	Paper waste: 40% Garden waste: 20% Organic waste: 15% Textile waste: 24% Wood waste: 43%	The decomposable fraction of degradable organic carbon in the different waste categories based on the wet bases	^51^
${k}_{j}$	Paper waste: 0.045 Garden waste: 0.065 Organic waste: 0.085 Textile waste: 0.045 Wood waste: 0.025	Based on the dry tropical climate where MAT—mean annual temperature > 20 °C and MAP—Mean annual precipitation < 1000mm reflecting the prevailing climatic conditions in Egypt	^51^

### Determination of energy production potentials

This section presents the estimation of potential energy production from each of the studied WtE projects (LFGE and waste incineration) The current study accounts for the maximum theoretical energy from WtE technologies that could potentially be retrieved from the total amount of wastes received at each governorate after deducting recycling and composting (10%), which leaves 90% of the total waste quantities to assess the maximum energy potential.

The potential energy production is mainly dependent on waste quantities received at both of the studied WtE technologies (LFGE and incineration). Accordingly, to obtain a comparable energy results, 20 years of operation would be applied for both WtE technologies.

### Estimation of LFGE potential energy

Quantity of the generated landfill gas (LFG) and particularly the percentage of methane in the LFG is greatly affecting the amount of energy potentially generated from landfill gas energy (LFGE) technology^27^. Accordingly, to estimate the amount methane generated (tonnes) from the scenario 1 of disposing MSW in managed landfill till it reaches the waste acceptance levels after 20 years, Eq. 2 above was used with changing some of input parameters to reflect the conditions of well managed landfills, including oxidation factor $(OX)$, which is equal to 0.1 for managed landfills covered with oxidizing material and methane correction factor ($MCF$), which is equal to 1.0 for anaerobic managed landfills^51^. Although, the CH_4_ emissions would be generated beyond the 20-years operation period, it has been assumed that regulatory arrangements or technology adaptations will manage LFG during that extended time through maintaining LFGE technology or implementing caps and closures to prevent uncontrolled CH_4_ releases.

Potential energy that could be produced from the combustion of methane content in the LFG was estimated using the Eq. 3 used by^14^, as shown below.

3 $${E}_{LFGE}=\frac{{LHV}_{{CH}_{4}}. {Q}_{{CH}_{4}} . {\varepsilon }_{ICE}. {\varepsilon }_{GC}}{3600}$$

where ${E}_{LFGE}$ is the potential energy production (MWh) from LFGE, $LHV$ is lower heating (calorific) value of methane, ${Q}_{{CH}_{4}}$ is the annual methane quantity (tonnes) acquired from the Eq. 2 above, ${\varepsilon }_{ICE}$ is the electrical energy conversion efficiency for the internal combustion engine (%) and ${\varepsilon }_{GC}$ is the landfill gas collection efficiency (%). Values used as input for the estimation of potential energy production from LFGE have been obtained from several literature as presented in Table 5.

**Table 5:** Values used in the estimation of potential energy production from LFGE.

Parameter	Value	References
$LHV$	37.2MJ/m^3^ (51.88 MJ⁄kg)	^14,27^
${\varepsilon }_{ICE}$	40%	^14,68^
${\varepsilon }_{GC}$	75%	^14^

The total potential energy generated from each governorate for the 20 years duration has been calculated using Eq. 4 below.

4 $${E}_{{T}_{LFGE}}=\sum_{x=1}^{y}{E}_{LFGE}$$

where ${E}_{{T}_{LFGE}}$ is the total potential energy production (MWh) from LFGE in specific governorate for 20 years, ${E}_{LFGE}$ is the potential energy production (MWh) from LFGE during specific modeling year, $x$ is the starting year for waste disposal, and $y$ is the final modeling year.

### Estimation of incineration potential energy

The main contributing factor in harnessing energy from waste incineration is the energy content of unit mass of the waste, which is called the total calorific value (kJ/kg) of waste^53^. Accordingly, to estimate potential energy (MWh) from waste incineration, the total calorific value of waste should firstly be estimated, as provided in Eq. 5 to be used in the calculation of potential power (kW) available from MSW incineration, as in Eq. 6^37^. Afterwards, potential electrical energy (MWh) has been calculated using Eq. 7, as obtained from^53^.

5 $${LCV}_{T}= \sum_{j=1}^{m}{LCV}_{j} . {F}_{{W}_{j}}$$

6 $${P}_{INC}= {LCV}_{T}. {\varepsilon }_{INC} . {W}_{q} . \frac{1000}{24 . 60 . 60}$$

7 $${E}_{INC}=\frac{{P}_{INC}. {C}_{f} . 8760}{1000}$$

where ${LCV}_{T}$ is the total calorific value of the MSW generated from each governorate (kJ/kg), ${LCV}_{j}$ is the lower calorific value of each waste fraction (kJ/kg), ${F}_{{W}_{j}}$ is the fraction of each type of MSW to be incinerated (%), as obtained in Table 3, ${P}_{INC}$ is the potential power available from MSW incineration (kW), ${\varepsilon }_{INC}$ is incineration electrical recovery efficiency (%), ${W}_{q}$ is the quantity of MSW per annum (tonnes/year), as obtained above from Eq. 1 above, ${E}_{INC}$ is the potential energy produced from MSW incineration (MWh/year), ${C}_{f}$ is the capacity factor and $8760$ is the number of hours per year. Values used as input for the estimation of potential energy production from incineration have been obtained from several literature as presented in Table 6.

**Table 6:** Values used in the estimation of potential energy production from incineration.

Parameter	Value	References
${LCV}_{j}$	Paper: 2729 kcal/kg Organic: 712 kcal/kg Textile: 1921 kcal/kg Wood: 2490 kcal/kg Plastics: 8193 kcal/kg	^36^
${\varepsilon }_{INC}$	30%	^53^
${C}_{f}$	75%	

The total potential energy generated from each governorate for the 20 years duration has been calculated using Eq. 8 below.

8 $${E}_{{T}_{INC}}=\sum_{x=1}^{y}{E}_{INC}$$

where ${E}_{{T}_{INC}}$ is the total potential energy production (MWh) from incineration in specific governorate for 20 years, ${E}_{INC}$ is the potential energy production (MWh) from incineration during specific modeling year, $x$ is the starting year for waste recipient, and $y$ is the final modeling year.

### Avoided GHG emissions

Implementing WtE projects would mitigate the impact of climate change by curbing/avoiding the emission of a large quantity of GHG to the atmosphere^54^. Through, firstly, preventing the release of CH_4_ emissions from open landfills and dumpsites that would otherwise occur without any intervention; and secondly, by generating electricity, which reduces the need for fossil fuel consumption typically required in conventional thermal power plants^14,55^. Certified Emission Reductions (CERs) resulting from the GHG avoidance/reduction could be traded on global markets, with prices fluctuating based on the specific market. For instance, the price per tonne of CO_2_ equivalent (tCO_2_e) ranges from $31 in California to $99 in the UK^56^.

To calculate the GHG avoided from open landfill/ dumpsites (${GHG}_{{Avoided }_{LF}}$) in t CO_2_ eq./year, it has been assumed that about 90% of the CH_4_ generated from those sites is released to the atmosphere, while the remaining 10% is oxidized near the surface of the landfill^38^. Equation 9 proposed by^27^ has been used.

9 $${GHG}_{{Avoided }_{LF}} = {Q}_{{CH}_{4}baseline} . {GWP}_{{CH}_{4}}. 0.9$$

where ${Q}_{{CH}_{4}baseline}$ is the annual methane quantity (tonnes) for baseline scenario provided above in Eq. 9 and ${GWP}_{{CH}_{4}}$ is the global warming potential of CH_4_, which is about 28 times the GWP of CO_2_, according to IPCC fifth assessment repot)^57^.

To estimate the avoided GHG emissions from electricity generation from any of the WtE technologies, Eq. 10 proposed by^55^ has been used, as follows:

10 $${GHG}_{{Avoided }_{WtE}}={{E}_{WtE}.Ef}_{grid}$$

where ${GHG}_{{Avoided }_{WtE}}$ is the avoided GHG emissions from electricity generation from WtE technology (either incineration or LFGE) (in t CO_2_ eq./year), ${E}_{{T}_{WtE}}$ is the annual energy production through WtE technology (kWh), ${Ef}_{grid}$ is the grid emission factor (kg CO_2_ eq./kWh). In the current study, grid emission factor of 0.51 kg CO_2_ eq./kWh has been used in the evaluation of avoided GHG emissions. The grid emission factor represents an average value of monthly emission factors for Egyptian electricity grid for seven consecutive years from 2016 to 2022, as has been acquired from Egyptian Electric Utility & Consumer Protection Regulatory Agency^58^.

Accordingly, the total avoided GHG emissions (${GHG}_{{Avoided }_{T}}$) (in t CO_2_ eq./year) from WtE technology has been estimated using the following Eq. 11.

11 $${GHG}_{{Avoided }_{T}} = {GHG}_{{Avoided }_{LF}}+ {GHG}_{{Avoided }_{WtE}}$$

### Assessment of economic feasibility

Understanding the economic viability of a project is crucial before making any investment decisions. The current study evaluated the economic viability of the two proposed WTE technologies—incineration and LFGE—through estimating the capital, operational, and maintenance expenditure following by evaluating several economic indicators like net present value (NPV) and, life cycle cost (LCC), levelized cost of energy (LCOE), internal rate of return (IRR), and payback period (PBP). The WtE project is deemed economically feasible when it has a positive NPV, signifying that the current value of cash inflows surpasses the current value of cash outflows. Additionally, when the IRR exceeds the discount rate, indicating that the project yields a return greater than the necessary rate of return, thus constituting a profitable investment^56^. Also, the project is deemed beneficial when the LCOE is less than the electricity FiT, providing the investor with an additional profit margin^40^.

The economic assessment assumes the following: (1) the lifetime of both WtE technologies is 20 years started from 2022 ending in 2041. (2) discount rate in 2020 of 9.25% was used, as per^36^. (3) exchange rate of 2020 was used in conversion from EGP to USD (USD = 15.73 EGP). (4) WtE projects would be able to trade carbon emission reductions (CERs) from the beginning of the projects, as Egyptian regulations allows the trading of CERs, accordingly, the revenue streams include the potential sale of tradable CERs. (5) price of CER is assumed to be 31 USD/tCO_2_e, as per^56^. (6) gate fee, which is a cost levied on a specific quantity of waste received by a waste treatment facility, are not considered in the revenue streams, as the existing regulations in Egypt does not support such revenue stream^36^. (7) all WtE facilities are assumed to be at the same distance from each other. Therefore, any changes in transportation due to the redirection of waste from WtE facility to another are considered to be insignificant.

### Estimation of capital and operation and maintenance costs

For a proposed LFGE plant, the initial investment cost ${Cost}_{inv}$ and operation and maintenance costs ${Cost}_{{O\&M}_{x}}$ are estimated according to Eqs. 12–18 obtained from^38^.

12 $${Cost}_{{inv}_{LFGE}}= {Cost}_{1}+ {Cost}_{2}+ {Cost}_{3}+ {Cost}_{4}+{Cost}_{5}$$

13 $${Cost}_{1}={W}_{n}x \$85 x [{W}_{d}(ft)-10\left(ft\right)]$$

14 $${Cost}_{2}= {W}_{n}x \$\text{1,700}$$

15 $${Cost}_{3}= {(Q}_{{CH}_{4}}{)}^{0.6}x \$\text{4,600}$$

16 $${Cost}_{4}= {W}_{n}x \$700$$

17 $${Cost}_{5}={(P}_{LFGE} x \$1300)+\$\text{1,100,000}$$

18 $${Cost}_{{O\&M}_{LFGE}}= {(E}_{LFGE} x \$0.025)+[\left({W}_{n}x \$\text{5,100}\right)+\$\text{5,100}]$$

where ${Cost}_{1}$ is the capital cost for installing vertical gas extraction wells, ${Cost}_{2}$ is the installation cost of wellheads and pipes, ${Cost}_{3}$ is the installation cost of knockout, blower, and flare system, ${Cost}_{4}$ is the cost of engineering, permitting, and surveying, ${Cost}_{5}$ is the installation cost of internal combustion engine (ICE), ${W}_{n}$ is the number of drilled wells which was taken as 50 after^40^, ${W}_{d}$ is the depth of the well which was taken as 65 ft according to^27^, ${Q}_{{CH}_{4}}$ is the methane flow rate (m^3^/year), ${P}_{LFGE}$ is the potential power of the LFGE plant (kW), and ${E}_{LFGE}$ is the annual energy production (kWh) of the LFGE plant.

On the other hand, for a proposed incineration plant, the initial investment cost ${Cost}_{inv}$ and operation and maintenance costs ${Cost}_{{O\&M}_{x}}$ are estimated according to Eqs. 19 and 20 obtained from^40^.

19 $${Cost}_{{inv}_{INC}}={(P}_{INC}{)}^{0.82} x \$16, 587$$

20 $${Cost}_{{O\&M}_{INC}}= {Cost}_{{inv}_{INC}} x 0.04$$

where ${Cost}_{{inv}_{INC}}$ the initial investment cost in incineration plant (USD), ${P}_{INC}$ is the potential power of the incineration plant (kW), and ${Cost}_{{O\&M}_{INC}}$ is operation and maintenance costs of incineration plant (USD).

### Economic indicators

The NP,V LCC, LCOE, IRR, and PBP of the proposed two technologies were estimated using Equations from 21 to 29 as obtained from^38,40,56^.

21 $$NPV= \sum_{n=1}^{y}\frac{{CF}_{n}}{{(1+{d}_{r})}^{n}}$$

22 $${CF}_{n}= {Rev}_{x}-({Cost}_{inv}+ {Cost}_{{O\&M}_{x}}+{Tax}_{x})$$

23 $${Rev}_{x}= {[E}_{WtE} x FiT]+[ {GHG}_{{Avoided }_{T}} x {CER}_{p}]$$

24 $${Tax}_{x}={P}_{{inv}_{x}} x {Tax}_{rate}$$

25 $${P}_{{inv}_{x}}={Rev}_{x}-{Cost}_{{O\&M}_{x}}$$

26 $$LLC= {Cost}_{inv}+\sum_{n=1}^{y}\frac{{Cost}_{{O\&M}_{n}}}{{(1+{d}_{r})}^{n}}$$

27 $$LCOE= \frac{LLC}{{E}_{WtE}}$$

28 $$IRR (the value of {d}_{r}that NPV= \sum_{n=0}^{N}\frac{{CF}_{n}}{{\left(1+{d}_{r}\right)}^{n}}=0$$

29 $$PBP= \frac{{Cost}_{inv}}{{Rev}_{x}-{Cost}_{{O\&M}_{x}}}$$

where $NPV$ is the net present value (USD), ${CF}_{n}$ is the net cash flow streams (USD), ${d}_{r}$ is the annual discount rate (%) which was taken as 9.25% after^36^, $n$ is the project lifetime (20 years),${Rev}_{x}$ is the revenue obtained from the WtE project, ${Cost}_{inv}$ is the initial investment cost (USD), ${Cost}_{{O\&M}_{x}}$ is operation and maintenance costs (USD), ${Tax}_{x}$ is the taxes paid on the profit of WtE project, ${E}_{WtE}$ the electricity generation potential of the WtE projects (kWh), $FiT$ is the Feed-in-Tariff which is 1.4 EGP/kWh (0.089 USD/kWh, based on 2020 exchange rate, the time of issuance of FiT regulations), ${GHG}_{{Avoided }_{T}}$ is the avoided GHG emissions from electricity generation from WtE technology, ${CER}_{p}$ is the price of tradable certified emission reduction certificate (USD/ tCO_2_e) which was taken as 31 USD/tCO_2_e after^56^, ${P}_{{inv}_{x}}$ is the profit of WtE project, ${Tax}_{rate}$ is the marginal tax rate of Egypt which was taken as 42.4%, as obtained from world bank data base^59^, $LCOE$ is the levelized cost of energy (USD/kWh), $LLC$ is the lifecycle cost (USD), $IRR$ is the internal rate of return, and $PBP$ is the payback period of the WtE project.

### Sensitivity analysis

Sensitivity analysis demonstrate how a particular system depends on defined input variables^38^. It’s vital to comprehend how changes in crucial economic variables could influence a project’s profitability. This knowledge is instrumental for decision-makers, enabling them to identify possible risks and opportunities for any prospective project^56^. Therefore, the economic viability of the proposed two WtE technologies was evaluated via an economic sensitivity analysis, taking into account variations in economic, technical, and policy aspects. Economic aspects include discount rate, Feed-in-Tariff (FiT), taxes, capital cost, operational and maintenance expenses, while technical aspects include the gas collection efficiency of LFGE, capacity factor of incineration technology, and electrical efficiency of WtE technology. Also, the policy aspects, proposed by decision-makers, include CER certificates and the inclusion of gate fee for WtE technologies. To assess the sensitivity of economic and technical aspects, a 10% rise and fall from their initially computed value was applied. For policy factors, varying values were employed as recommended by various researchers. The comparison of CER prices was made at 5 USD/tCO_2_e and 99 USD/tCO_2_e against the base value of 31 USD/tCO_2_e used in calculations^56^. Since the gate fee aspect is not yet in place in Egypt, the sensitivity of its inclusion in the revenue streams was examined using two values of 50–100 USD/tonne of received MSW, according to^19,60^.

## Results and discussion

This section discusses the MSW quantities generated from the selected six Egyptian governorates, baseline methane emissions, and potential energy production from WtE technologies (LFGE and incineration). Tables 7 and 8 includes summary of the results of the current research.

**Table 7:** Quantities of MSW (tonnes) generated at the studied governorates over 20 years.

Year	Giza	Minya	Qalubia	Sharqia	Sohag	Suez
2022	1,960,109	1,016,778	1,504,061	1,462,899	851,004	145,858
2023	2,059,214	1,068,187	1,580,108	1,536,864	894,031	153,233
2024	2,163,330	1,122,196	1,660,000	1,614,570	939,234	160,981
2025	2,272,710	1,178,935	1,743,931	1,696,204	986,723	169,120
2026	2,387,620	1,238,543	1,832,106	1,781,966	1,036,613	177,671
2027	2,508,341	1,301,165	1,924,739	1,872,064	1,089,025	186,654
2028	2,635,165	1,366,953	2,022,056	1,966,717	1,144,087	196,091
2029	2,768,402	1,436,068	2,124,293	2,066,156	1,201,933	206,006
2030	2,908,375	1,508,677	2,231,699	2,170,623	1,262,704	216,422
2031	3,055,425	1,584,957	2,344,536	2,280,372	1,326,548	227,364
2032	3,209,910	1,665,094	2,463,078	2,395,670	1,393,619	238,860
2033	3,372,207	1,749,283	2,587,614	2,516,798	1,464,082	250,937
2034	3,542,709	1,837,728	2,718,447	2,644,049	1,538,108	263,625
2035	3,721,832	1,930,646	2,855,894	2,777,735	1,615,876	276,954
2036	3,910,011	2,028,261	3,000,291	2,918,180	1,697,576	290,957
2037	4,107,705	2,130,812	3,151,988	3,065,726	1,783,407	305,668
2038	4,315,395	2,238,548	3,311,356	3,220,733	1,873,578	321,123
2039	4,533,586	2,351,731	3,478,782	3,383,576	1,968,308	337,359
2040	4,762,808	2,470,637	3,654,672	3,554,653	2,067,828	354,416
2041	5,003,621	2,595,555	3,839,456	3,734,380	2,172,379	372,336
Annual average (tonnes)	3,259,924	1,691,038	2,501,455	2,432,997	1,415,333	242,582
Total (tonnes)	65,198,473	33,820,753	50,029,109	48,659,935	28,306,662	4,851,636

**Table 8:** results of methane emissions and WtE total potential energy at the studied governorates.

	Giza	Minya	Qalubia	Sharqia	Sohag	Suez
Baseline total CH_4_ quantities (Mt)	1.05	0.55	0.81	0.78	0.46	0.08
Baseline (t CH_4_/t MSW)	0.02	0.02	0.02	0.02	0.02	0.02
LFGE total CH4 (Mt)	1.58	1.08	1.70	1.25	0.85	0.12
LFGE (t CH_4_/t MSW)	0.024	0.032	0.034	0.026	0.030	0.025
LFGE total energy (TWh)	3.01	2.05	3.0	2.27	1.49	0.22
LFGE (kWh/kg MSW)	0.046	0.061	0.060	0.047	0.053	0.045
MSW total LCV (MJ/t)	7,210	5,125	8,655	9,216	8,981	8,104
Incineration total energy (TWh)	29.38	10.83	27.06	28.03	15.89	2.46
Incineration (kWh/kg MSW)	0.451	0.320	0.541	0.576	0.561	0.507

### MSW quantities at the studied governorates

**Fig. 3:**
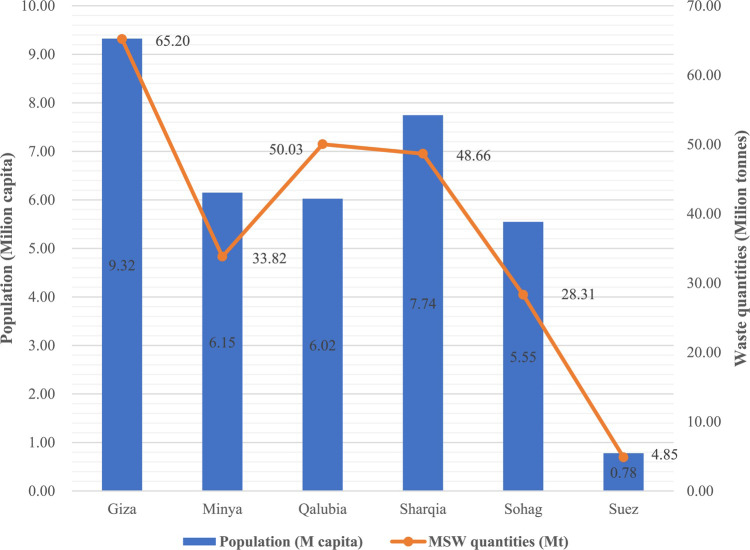
Population and waste quantities at the studied governorates.

The quantities of MSW potentially generated in each governorate were estimated over a 20 years period (2022–2041), reflecting the waste acceptance limit of landfills, as shown in Table 7. Figure 3 depicts the MSW quantities and population in each governorate.

Generally, waste quantity generated in each governorate is influenced by population size, waste generation rate, annual population growth rate, and annual GDP growth rate. Given that the annual population and GDP growth rates are consistent across Egypt, the primary variables affecting waste quantities are population size and waste generation rate. In specific, Giza governorate, with the highest population of 9.3 million, generated the most MSW over 20 years, totaling 65.2 million tonnes, as shown in Fig. 3. Interestingly, Qalubia, despite being fourth in population size, generated the second highest amount of MSW (50.1 million tonnes) due to it has the highest waste generation rate of 0.760 kg/capita/day. This trend aligns with findings from previous studies^38^, which highlight the correlation between MSW quantities, population and generation rate. Our findings, in theory, highlight the importance of data availability regarding population size and waste generation rate in predicting MSW quantities. Practically, they highlight the need for tailored waste management strategies in governorates with high waste generation rates to mitigate the environmental impact.

### Baseline methane emissions

**Fig. 4:**
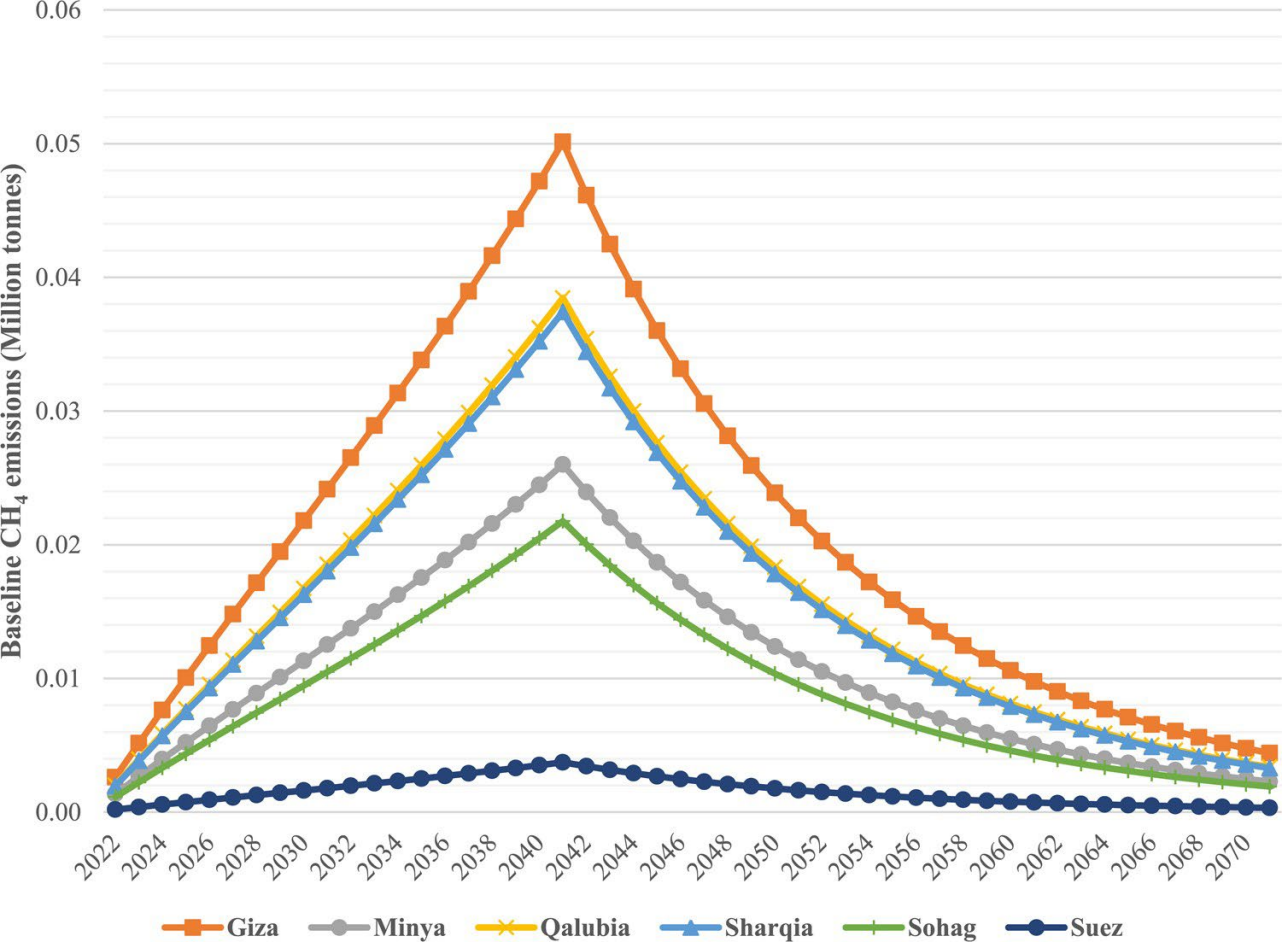
Baseline CH_4_ emissions from each governorate for 50 years period.

**Fig. 5:**
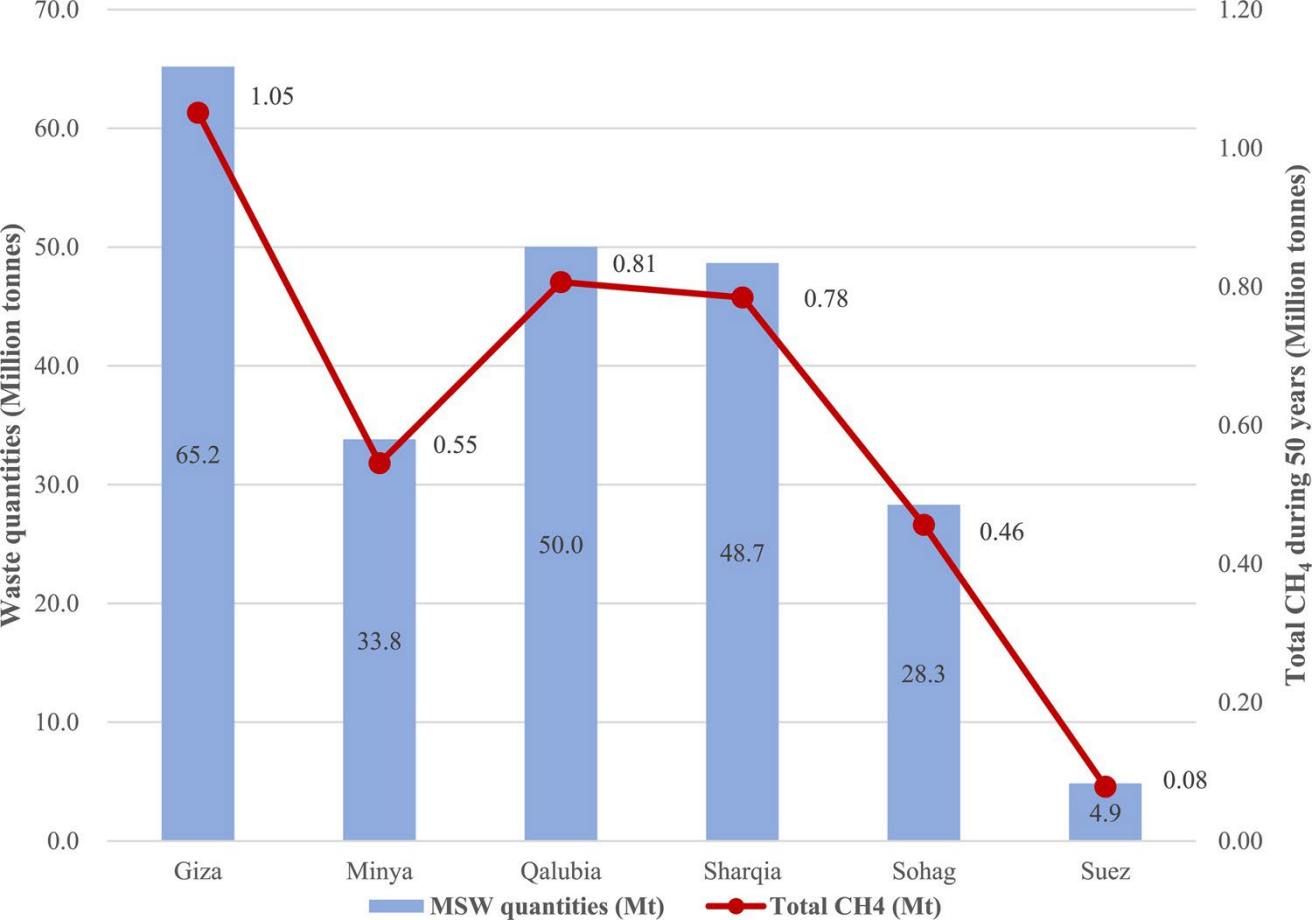
Baseline CH_4_ with relevant to MSW quantities.

The baseline CH_4_ emissions from each governorate were estimated over a 50 years period (2022–2072), covering the landfills’ operational life (20 years) and the subsequent 30 years at which CH_4_ continues to be emitted from the landfills. In general, Fig. 4 shows the baseline CH_4_ emissions for each governorate, which steadily increas until the year following landfill closure (2041) and then decline to minimal levels by 2072. This emission is consistent with previous studies^17,24,55,61^. In more detail, Fig. 5 compares the total CH_4_ emissions over 50 years with the generated waste quantities in each governorate. The results reveal that the total CH_4_ emissions are primarily dependent on waste quantities generated at each governorate, with minimal correlation with the percentage of DOC. This can be attributed to the conditions of open dumpsites/ unmanaged landfills, which lack a capping layer, thereby limiting the biodegradation of decomposable fractions of MSW. Specifically, Giza governorate showed the highest baseline CH_4_ emissions over 50 years (1.05 Mt CH_4_), followed by Qalubia (0.81 Mt CH_4_), Sharqia (0.78 Mt CH_4_), Minya (0.55 Mt CH_4_), Sohag (0.46 Mt CH_4_) and Suze (0.08 Mt CH_4_). The total baseline methane emissions from all governorates amounted to about 3.73 Mt CH_4_ over 50 years, with about 0.02 tonne of CH_4_ emitted per tonne of MSW, as shown in Table 8. Our findings align with the results of previous studies, such as^5^, which reported similar CH_4_ emission rates per tonne of MSW. Theoretically, our findings underscore the importance of waste quantity in predicting CH_4_ emissions, while practically, they highlight the need for improved landfill management practices to mitigate CH_4_ emissions.

### Energy production potentials from WtE technologies

#### LFGE potential energy

**Fig. 6:**
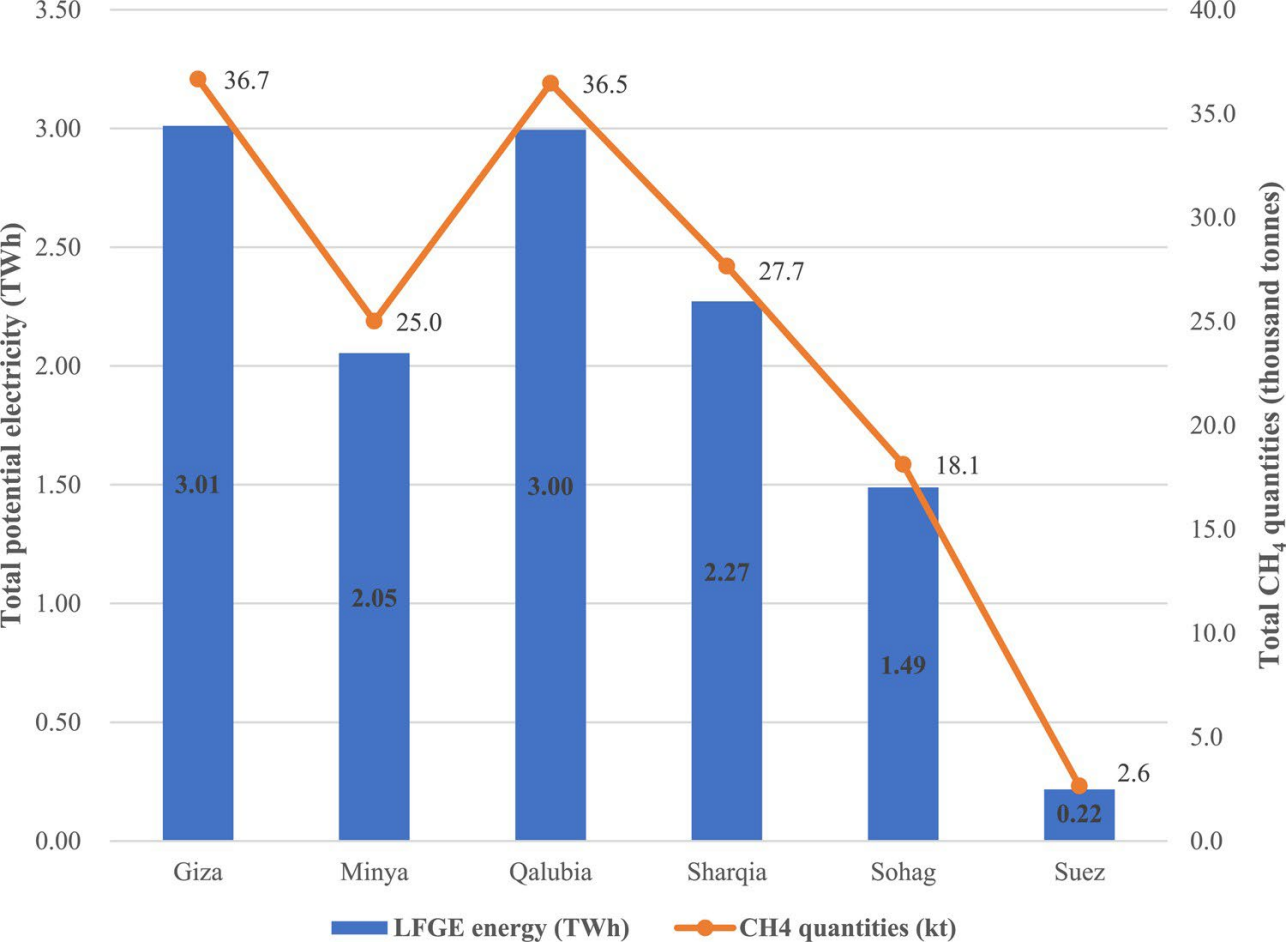
Total LFGE potential energy with relevant to the total CH_4_.

The total potential energy generated from methane quantities emitted from managed landfills was estimated over a of 20 years period (2022–2041). Figure 6 generally links the total potential energy produced from LFGE with the total generated CH_4_ in each governorate. The results show a direct relationship between the total energy potentially produced from LFGE and CH_4_ quantities, as expected from Eq. 3 above and consistent with the findings of^14,38^. Specifically, Giza showed the highest potential energy produced for the 20 years period followed by Qalubia, Sharqia, Minya, Sohag and Suez with a total energy of 3.01, 3.0, 2.27, 2.05, 1.49 and 0.22 TWh, respectively. As indicated in Table 3, the annual electricity consumption for all studied governorates amounted to 56.6 TWh annually, based on a per capita consumption of 1592 kWh/capita^69^. Accordingly, implementing LFGE technology in all governorates has the potential to produce aggregated energy amounting to about 12.1 TWh throughout 20 years, which corresponds to about 0.6 TWh produced annually, representing about 1% of the total electricity consumption of all governorates.

Table 8 revealed that the amount of energy generated from LFGE relevant to the quantities of MSW in each governorate ranged from 0.045 to 0.061 kWh/kg MSW, which are relatively lower than the results obtained from^15,61^ of 0.12 kWh/kg MSW and that from^16^ of 0.37 kWh/kg MSW. The differences can be primarily attributed to varying methane generation rates and subsequently, the amount of generated electricity obtained in each study. Climatic conditions also play a significant role: Egypt’s dry arid weather contrasts with the wet tropical conditions in the other studies, favoring the decomposition of MSW’s organic degradable fractions and resulting in higher CH_4_ quantities, as per^51^. Additionally, different modeling techniques used in the determination of CH_4_ emissions as noted by^47^, can lead to varying results. These results carry important implications both theoretically and practically. From a theoretical perspective, they emphasize the critical role of methane generation rates and climatic conditions in forecasting energy output from LFGE. Practically, they demonstrate that LFGE has a low potential for contributing a significant amount to the energy mix of the studied governorates.

#### Incineration potential energy

**Fig. 7:**
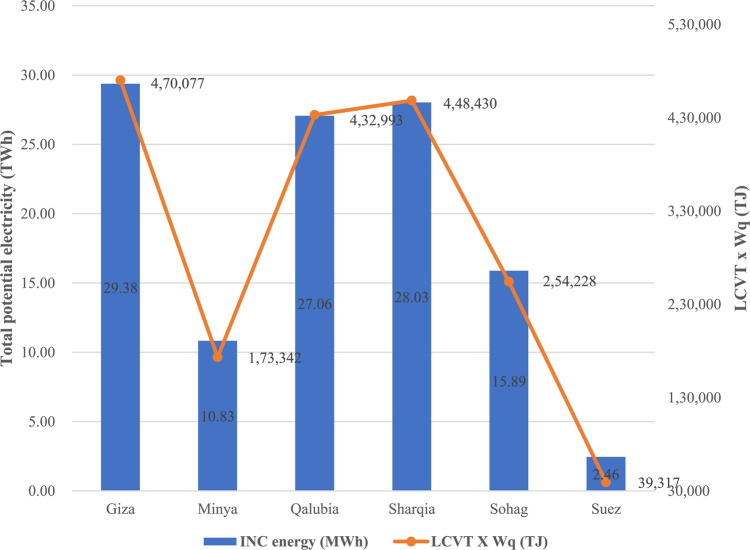
Total incineration potential energy.

The total potential energy generated from MSW incineration was estimated for a of 20 years period (2022–2041), corresponding to the period during which the landfills received MSW from each governorate. Figure 7 shows the total potential energy produced from MSW incineration in the studied governorates. Generally, the Figure indicates that the total energy potentially produced from incineration is directly proportional to the result of multiplication of the total calorific value (LCV_T_) of the MSW and the total waste quantities (W_q_) generated from each governorate, as expected from Eqs. 6 and 7 above and consistent with the findings of^15^. Specifically, Giza indicated the highest potential energy from incineration followed by Sharqia, Qalubia, Sohag, Minya, and Suez with a total energy of 29.4, 28.1, 27.1, 15.8, 10.8, and 2.4 TWh, respectively, over the 20 years period. As mentioned earlier, the annual electricity consumption for all studied governorates amounted to 56.6 TWh annually, as shown in Table 3. Implementing incineration technology in all governorates has the potential to produce aggregated energy that would amount to about 113.6 TWh over 20 years, which corresponds to about 5.6 TWh produced annually, representing about 10% of the total electricity consumption of all governorates. The LCV_T_ values indicated that almost all governorates, except Minya, have the potential to produce viable energy from incineration as they were within and exceeding the viable range of LCV_T_ claimed by^15,31^ which is from 6000 to 7000 kJ/kg MSW. Table 8 reveales that the amount of energy generated from incineration relevant to the quantities of MSW in each governorate ranged from 0.32 to 0.57 kWh/ kg MSW, which aligns well with the results of^15,16,41^, which ranged from 0.45 to 0.56 kWh/ kg MSW. These findings have important implications. Theoretically, they emphasize the significance of calorific value and waste quantities in predicting energy output from MSW incineration. Practically, they highlight the potential of incineration technology to significantly contribute to the energy mix at the studied governorates.

### Avoided GHG emissions

The total GHG emissions that could be avoided as a result of implementing WtE project was estimated a time period of 20 years, as shown in Table 9. The results indicate that Giza has the highest potential to avoid GHG emissions from either LFGE or incineration technologies. For instance, implementing LFGE project in Giza could avoid about 696,422 tonnes of CO_2_ equivalent annually (13.2 Mt CO_2_ eq. for 20 years), while it could avoid approximately double that figure, with about 1,331,590 tonnes of CO_2_ equivalent could be avoided annually (26.6 Mt CO_2_ eq. over 20 years). This is basically due to the high energy production from incineration in comparison to LFGE technology, which avoids the GHG emissions from the use of fossil fuel typically required in conventional thermal power plants. This trend is consistent across other governorates, where the GHG emissions avoided through incineration are approximately double those avoided through LFGE. Accordingly, if decision-makers opt for implementing LFGE in all governorates, this option would avoid about 2.5 Mt CO_2_ eq. annually (47 Mt CO_2_ eq. over 20 years). Alternatively, implementing incineration technology would avoid about 5 Mt CO_2_ eq. annually (99 Mt CO_2_ eq. over 20 years). From a theoretical standpoint, our results highlight the necessity of evaluating various WtE technologies for effective GHG mitigation. Practically, they demonstrate the considerable potential of incineration technology, over LFGE, in reducing GHG emissions, emphasizing the importance of strategic planning to maximize environmental benefits.

**Table 9:** Avoided GHG emissions from the implementation of WtE projects (in t CO_2_ eq./Y).

	Giza		Minya		Qalubia		Sharqia		Sohag		Suez	
Year	LFGE avoided GHG	INC avoided GHG	LFGE avoided GHG	INC avoided GHG	LFGE avoided GHG	INC avoided GHG	LFGE avoided GHG	INC avoided GHG	LFGE avoided GHG	INC avoided GHG	LFGE avoided GHG	INC avoided GHG
2022		448,853		165,516		413,444		428,184		242,750		37,542
2023	74,601	537,521	40,087	208,107	58,855	484,971	55,591	499,071	32,704	283,667	5,519	44,349
2024	147,014	625,402	79,003	250,118	116,056	556,072	109,584	569,610	64,491	324,364	10,879	51,109
2025	217,611	712,881	116,946	291,740	171,887	627,050	162,252	640,099	95,519	365,016	16,107	57,850
2026	286,740	800,329	154,105	333,156	226,620	698,198	213,852	710,828	125,939	405,789	21,229	64,600
2027	354,733	888,103	190,657	374,541	280,513	769,801	264,631	782,076	155,893	446,846	26,269	71,386
2028	421,905	976,549	226,772	416,065	333,811	842,134	314,822	854,115	185,518	488,343	31,250	78,236
2029	488,557	1,066,003	262,610	457,892	386,748	915,468	364,648	927,213	214,944	530,435	36,194	85,174
2030	554,979	1,156,796	298,327	500,183	439,550	990,065	414,323	1,001,630	244,295	573,273	41,123	92,226
2031	621,447	1,249,251	334,071	543,092	492,433	1,066,188	464,052	1,077,623	273,693	617,005	46,057	99,416
2032	688,231	1,343,688	369,988	586,774	545,606	1,144,092	514,036	1,155,449	303,253	661,779	51,016	106,770
2033	755,591	1,440,422	406,216	631,379	599,274	1,224,035	564,467	1,235,362	333,089	707,741	56,019	114,311
2034	823,780	1,539,769	442,893	677,058	653,636	1,306,271	615,534	1,317,614	363,311	755,038	61,085	122,064
2035	893,047	1,642,044	480,151	723,960	708,887	1,391,058	667,421	1,402,461	394,028	803,817	66,232	130,053
2036	963,637	1,747,562	518,122	772,233	765,218	1,478,653	720,312	1,490,160	425,346	854,225	71,478	138,303
2037	1,035,792	1,856,643	556,935	822,027	822,821	1,569,315	774,384	1,580,969	457,371	906,412	76,842	146,838
2038	1,109,749	1,969,607	596,719	873,492	881,883	1,663,310	829,818	1,675,152	490,207	960,530	82,340	155,682
2039	1,185,749	2,086,782	637,603	926,781	942,593	1,760,906	886,790	1,772,978	523,961	1,016,732	87,991	164,863
2040	1,264,030	2,208,500	679,715	982,048	1,005,140	1,862,377	945,479	1,874,718	558,735	1,075,176	93,811	174,405
2041	1,344,833	2,335,102	723,184	1,039,450	1,069,713	1,968,003	1,006,064	1,980,655	594,636	1,136,024	99,820	184,334
Average	696,422	1,331,590	374,427	578,781	552,697	1,136,571	520,424	1,148,798	307,207	657,748	51,645	105,976
Total	13,232,026	26,631,809	7,114,104	11,575,612	10,501,246	22,731,411	9,888,060	22,975,967	5,836,933	13,154,963	981,262	2,119,511

### Economic feasibility

This study utilizes the results of five key economic indicators (NPV, LCC, LCOE, IRR, and PBP) to evaluate the economic feasibility of the proposed WtE technologies for the governorates under investigation, as displayed in Table 10.

**Table 10:** The economic feasibility of two WtE technologies in the studied governorates projected over a 20-year.

Economic indicators	Unit	Giza		Minya		Qalubia		Sharqia		Sohag		Suez	
		LFGE	INC	LFGE	INC	LFGE	INC	LFGE	INC	LFGE	INC	LFGE	INC
Capital Cost	M$	220	348	173	154	219	325	184	335	141	210	44	45
O&M Cost	M$	4	14	3	6	4	13	3	13	2	8	0	2
NPV	M$	− 394	− 120	− 331	− 94	− 407	− 131	− 339	− 134	− 273	− 123	− 93	− 49
LCC	M$	222	354	174	156	221	331	185	341	142	214	44	46
LCOE	USD/kWh	1.40	0.24	1.61	0.29	1.40	0.24	1.55	0.24	1.81	0.27	3.86	0.38
IRR	%	− 1%	16%	− 5%	13%	− 3%	16%	− 3%	16%	− 6%	14%	− 18%	8%
PBP	Years	7.01	2.24	9.45	2.60	8.15	2.32	7.83	2.32	9.85	2.59	20.10	3.73

*NPV Analysis*: The NPV for both WtE technologies across all governorates was found to be negative, with values ranging from -93 million USD for LFGE in Suez to − 407 million USD in Qalubia. This negative NPV indicates that the projected income does not outweigh the initial investment cost, making these projects economically unfeasible under the current assumptions.

*IRR Analysis*: In terms of IRR, LFGE technology across all governorates returned negative percentages, ranging from -1% in Giza to -18% in Suez, indicating that the LFGE projects yield a return lower than the required rate of return. Conversely, incineration technology returned positive IRR values higher than the discount rate across all governorates, ranging from 13 to 16%, except for Suez which had an IRR of 8%. The higher return rate of incineration technology can be attributed to the fact that it has higher projected cash inflows due to higher energy production, leading to more revenue from electricity sales.

*LCOE Analysis*: For LCOE, WtE technologies across all governorates could not offer a cost of electricity generation that would provide a beneficial margin when compared with the Egyptian electricity FiT of 0.089 USD/kWh. The LCOE of WtE technologies exceeds the FiT, with values ranging between 1.40 USD/ kWh in Giza and Qalubia to 3.86 USD/kWh in Suez for the LFGE technology, and between 0.24 USD/kWh in Giza, Qalubia, and Sharqia to 0.38 USD/kWh in Suez for the incineration technology. This is primarily due to the high initial investment costs associated with WtE technologies.

*PBP Analysis*: In terms of the payback period, the PBP for incineration technology is generally lower than that for LFGE technology across all governorates, given the higher potential revenue from incineration due to its higher energy production.

*Overall Implications*: Overall, the results indicated that all proposed WtE technologies are not economically viable in all studied governorates under the assumptions made. However, incineration technology could have a slight advantage over LFGE as some of its economic indicators have positive values, such as IRR and PBP. These results are in line with economic results obtained in similar studies conducted in developing economies like Brazil and China, as reported by^53^. Furthermore, these findings align with the reality that the existing FiT regulation doesn’t provide a competitive FiT rate, and the gate fee, a significant revenue element for these projects, is not considered in the regulation^36^. From a theoretical standpoint, our results underscore the importance of comprehensive economic evaluations in assessing the feasibility of WtE technologies. Practically, they highlight the need for policy adjustments, such as more competitive FiT rates and the inclusion of gate fees, to improve the economic viability of WtE projects.

### Sensitivity analysis

Sensitivity analysis was carried out by altering various technical, economic factors, as well as policy factors to evaluate their influence on the profitability of the project.

#### Economic factors

The outcomes of the sensitivity analysis conducted for economic factors, including the discount rate, FiT, taxes, capital cost, operational and maintenance expenses are presented in Table 11. This analysis was performed using a 10% deviation (both increase and decrease) from their initially computed values.

**Table 11:** Results of sensitivity analysis conducted for economic factors.

		NPV (M$)		IRR (%)		LCC (M$)		LCOE ($/kWh)		PBP (Y)	
		LFGE	INC	LFGE	INC	LFGE	INC	LFGE	INC	LFGE	INC
		Giza									
Base value		− 393.80	− 120.14	− 1.48%	16.02%	221.55	354.52	1.398	0.241	7.011	2.242
Discount rate	− 10%	− 389.55	− 69.05	− 1.48%	16.02%	221.68	354.52	1.399	0.241	7.016	2.242
	+ 10%	− 397.20	− 164.92	− 1.48%	16.02%	221.43	353.70	1.397	0.241	7.008	2.237
Feed-in-Tarrif	− 10%	− 398.28	− 173.80	− 1.98%	14.52%	221.55	354.09	1.398	0.241	7.339	2.441
	+ 10%	− 389.32	− 66.49	− 1.01%	17.47%	221.55	354.09	1.398	0.241	6.712	2.068
Taxes	− 10%	− 385.52	− 69.43	− 0.63%	17.37%	221.55	354.09	1.398	0.241	7.011	2.240
	+ 10%	− 402.08	− 170.85	− 2.42%	14.62%	221.55	354.09	1.398	0.241	7.011	2.240
Capital cost	− 10%	− 343.18	− 38.91	− 0.22%	18.05%	199.57	318.68	1.259	0.217	6.316	1.998
	+ 10%	− 444.43	− 201.37	− 2.66%	14.28%	243.53	389.50	1.537	0.265	7.707	2.485
Operational cost	− 10%	− 393.71	− 119.81	− 1.47%	16.03%	221.37	354.71	1.397	0.241	6.916	2.216
	+ 10%	− 393.90	− 120.47	− 1.49%	16.01%	221.72	353.46	1.399	0.241	7.109	2.263
		Minya									
Base value		− 330.63	− 94.13	− 5.05%	13.36%	173.75	156.26	1.607	0.288	9.447	2.604
Discount rate	− 10%	− 329.81	− 75.24	− 5.05%	13.36%	173.84	156.44	1.608	0.289	9.452	2.607
	+ 10%	− 331.09	− 110.64	− 5.05%	13.36%	173.67	156.08	1.606	0.288	9.442	2.601
Feed-in-Tarrif	− 10%	− 333.69	− 113.91	− 5.70%	12.04%	173.75	156.26	1.607	0.288	9.968	2.831
	+ 10%	− 327.58	− 74.34	− 4.45%	14.63%	173.75	156.26	1.607	0.288	8.977	2.410
Taxes	− 10%	− 325.72	− 74.78	− 4.10%	14.59%	173.75	156.26	1.607	0.288	9.447	2.604
	+ 10%	− 335.55	− 113.48	− 6.13%	12.08%	173.75	156.26	1.607	0.288	9.447	2.604
Capital cost	− 10%	− 290.89	− 58.28	− 3.65%	15.21%	156.50	140.63	1.447	0.260	8.509	2.320
	+ 10%	− 370.38	− 129.97	− 6.40%	11.77%	191.00	171.88	1.766	0.317	10.385	2.894
Operational cost	− 10%	− 330.57	− 93.98	− 5.04%	13.37%	173.63	155.98	1.606	0.288	9.297	2.573
	+ 10%	− 330.70	− 94.27	− 5.06%	13.35%	173.87	156.53	1.608	0.289	9.602	2.635
		Qalubia									
Base value		− 406.79	− 131.14	− 3.12%	15.51%	220.79	331.01	1.401	0.245	8.151	2.320
Discount rate	− 10%	− 404.19	− 85.36	− 3.12%	15.51%	220.92	331.42	1.401	0.245	8.156	2.323
	+ 10%	− 408.73	− 171.26	− 3.12%	15.51%	220.67	330.65	1.400	0.244	8.146	2.318
Feed-in-Tarrif	− 10%	− 411.22	− 180.56	− 3.72%	14.01%	220.79	331.01	1.401	0.245	8.596	2.534
	+ 10%	− 402.36	− 81.72	− 2.57%	16.96%	220.79	331.01	1.401	0.245	7.749	2.140
Taxes	− 10%	− 399.59	− 85.12	− 2.23%	16.84%	220.79	331.01	1.401	0.245	8.151	2.320
	+ 10%	− 413.98	− 177.16	− 4.11%	14.13%	220.79	331.01	1.401	0.245	8.151	2.320
Capital cost	− 10%	− 356.33	− 55.20	− 1.81%	17.52%	198.88	297.91	1.262	0.220	7.342	2.069
	+ 10%	− 457.24	− 207.08	− 4.36%	13.78%	242.69	364.12	1.540	0.269	8.959	2.576
Operational cost	− 10%	− 406.70	− 130.83	− 3.11%	15.52%	220.61	330.43	1.399	0.244	8.024	2.295
	+ 10%	− 406.88	− 131.45	− 3.13%	15.50%	220.96	331.60	1.402	0.245	8.282	2.346
		Sharqia									
Base value		− 339.26	− 133.81	− 2.82%	15.55%	185.18	340.66	1.549	0.243	7.830	2.318
Discount rate	− 10%	− 336.84	− 86.65	− 2.82%	15.55%	185.28	341.07	1.549	0.243	7.834	2.321
	+ 10%	− 341.10	− 175.13	− 2.82%	15.55%	185.09	340.29	1.548	0.243	7.826	2.316
Feed-in-Tarrif	− 10%	− 342.63	− 184.99	− 3.34%	14.04%	185.18	340.66	1.549	0.243	8.199	2.533
	+ 10%	− 335.89	− 82.62	− 2.33%	17.02%	185.18	340.66	1.549	0.243	7.492	2.137
Taxes	− 10%	− 333.06	− 86.36	− 1.94%	16.89%	185.18	340.66	1.549	0.243	7.830	2.318
	+ 10%	− 345.46	− 181.25	− 3.80%	14.17%	185.18	340.66	1.549	0.243	7.830	2.318
Capital cost	− 10%	− 296.91	− 55.66	− 1.52%	17.57%	166.80	306.60	1.395	0.219	7.052	2.068
	+ 10%	− 381.61	− 211.96	− 4.05%	13.82%	203.57	374.73	1.702	0.267	8.607	2.574
Operational cost	− 10%	− 339.19	− 133.49	− 2.82%	15.56%	185.05	340.06	1.547	0.243	7.722	2.293
	+ 10%	− 339.33	− 134.12	− 2.83%	15.54%	185.32	341.26	1.550	0.244	7.940	2.344
		Sohag									
Base value		− 273.04	− 122.84	− 5.73%	13.73%	141.90	213.90	1.811	0.269	9.852	2.587
Discount rate	− 10%	− 272.69	− 96.86	− 5.73%	13.73%	141.97	214.16	1.812	0.270	9.857	2.590
	+ 10%	− 273.14	− 145.58	− 5.73%	13.73%	141.84	213.67	1.810	0.269	9.848	2.584
Feed-in-Tarrif	− 10%	− 275.24	− 151.86	− 6.35%	12.31%	141.90	213.90	1.811	0.269	10.354	2.829
	+ 10%	− 270.84	− 93.82	− 5.15%	15.11%	141.90	213.90	1.811	0.269	9.397	2.383
Taxes	− 10%	− 269.23	− 95.91	− 4.75%	15.00%	141.90	213.90	1.811	0.269	9.852	2.587
	+ 10%	− 276.85	− 149.77	− 6.84%	12.42%	141.90	213.90	1.811	0.269	9.852	2.587
Capital cost	− 10%	− 240.56	− 73.77	− 4.29%	15.64%	127.80	192.51	1.631	0.242	8.873	2.305
	+ 10%	− 305.52	− 171.91	− 7.12%	12.09%	156.00	235.29	1.991	0.296	10.831	2.875
Operational cost	− 10%	− 273.00	− 122.64	− 5.72%	13.74%	141.81	213.53	1.810	0.269	9.705	2.556
	+ 10%	− 273.09	− 123.04	− 5.74%	13.72%	141.99	214.28	1.812	0.270	10.004	2.618
		Suez									
Base value		− 93.16	− 48.86	− 18.00%	8.36%	44.19	46.29	3.862	0.377	20.102	3.733
Discount rate	− 10%	− 93.99	− 45.32	− 18.00%	8.36%	44.21	46.35	3.863	0.377	20.108	3.737
	+ 10%	− 92.37	− 51.94	− 18.00%	8.36%	44.18	46.24	3.860	0.376	20.097	3.729
Feed-in-Tarrif	− 10%	− 93.48	− 53.35	− 19.90%	7.14%	44.19	46.29	3.862	0.377	21.079	4.094
	+ 10%	− 92.84	− 44.37	− 16.17%	9.53%	44.19	46.29	3.862	0.377	19.212	3.430
Taxes	− 10%	− 92.56	− 44.67	− 14.90%	9.46%	44.19	46.29	3.862	0.377	20.102	3.733
	+ 10%	− 93.76	− 53.05	− 21.40%	7.22%	44.19	46.29	3.862	0.377	20.102	3.733
Capital cost	− 10%	− 83.02	− 38.24	− 15.45%	10.00%	39.79	41.66	3.477	0.339	18.100	3.311
	+ 10%	− 103.30	− 59.48	− 22.36%	6.93%	48.59	50.92	4.246	0.414	22.104	4.167
Operational cost	− 10%	− 93.15	− 48.82	− 17.96%	8.37%	44.17	46.21	3.860	0.376	19.716	3.672
	+ 10%	− 93.17	− 48.90	− 18.03%	8.35%	44.21	46.37	3.863	0.377	20.503	3.795

**Fig. 8:**
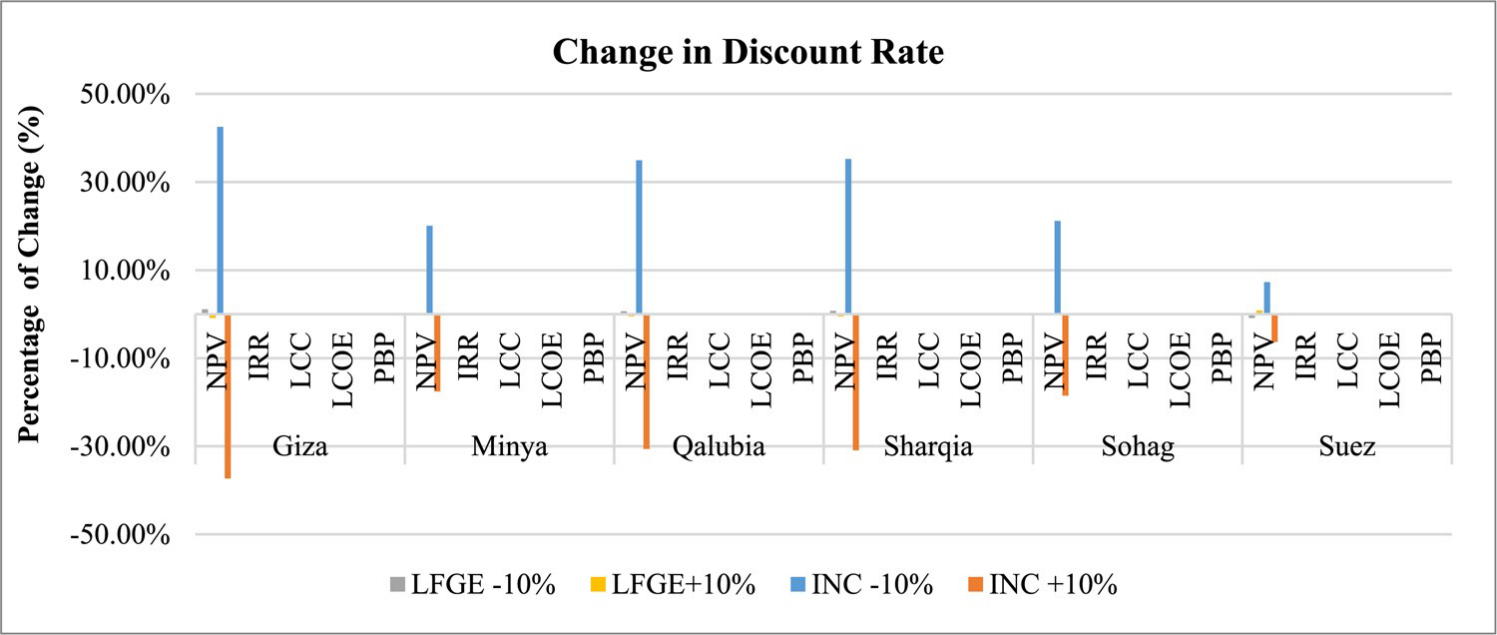
Sensitivity of changing discount rate in WtE profitability.

*Discount Rate Sensitivity*: Fig. 8 depicts the sensitivity of changing the discount rate from its original value of 9.25% on the different economic factors of the WtE projects. The analysis generally indicates a direct correlation between the NPV of incineration projects and the discount rate, with a decrease in the discount rate favorably impacting the NPV. Specifically, a 10% decrease in the discount rate results in a significant increase in the NPV of incineration projects across all governorates, ranging between a 7% increase in Suez to a 43% increase in Giza. Conversely, a 10% increase in the discount rate substantially reduces the NPV of incineration projects across all governorates, with reductions ranging between 6% in Suez to 37% in Giza. This sensitivity can be attributed to the higher projected cash inflows from incineration projects due to their higher energy production compared to LFGE projects. Other economic indicators (LCC, LCOE, IRR, and PBP) do not show significant sensitivity to changes in the discount rate. For LFGE projects, changes in discount rates do not show significant impact on all economic indicators across all governorates.

**Fig. 9:**
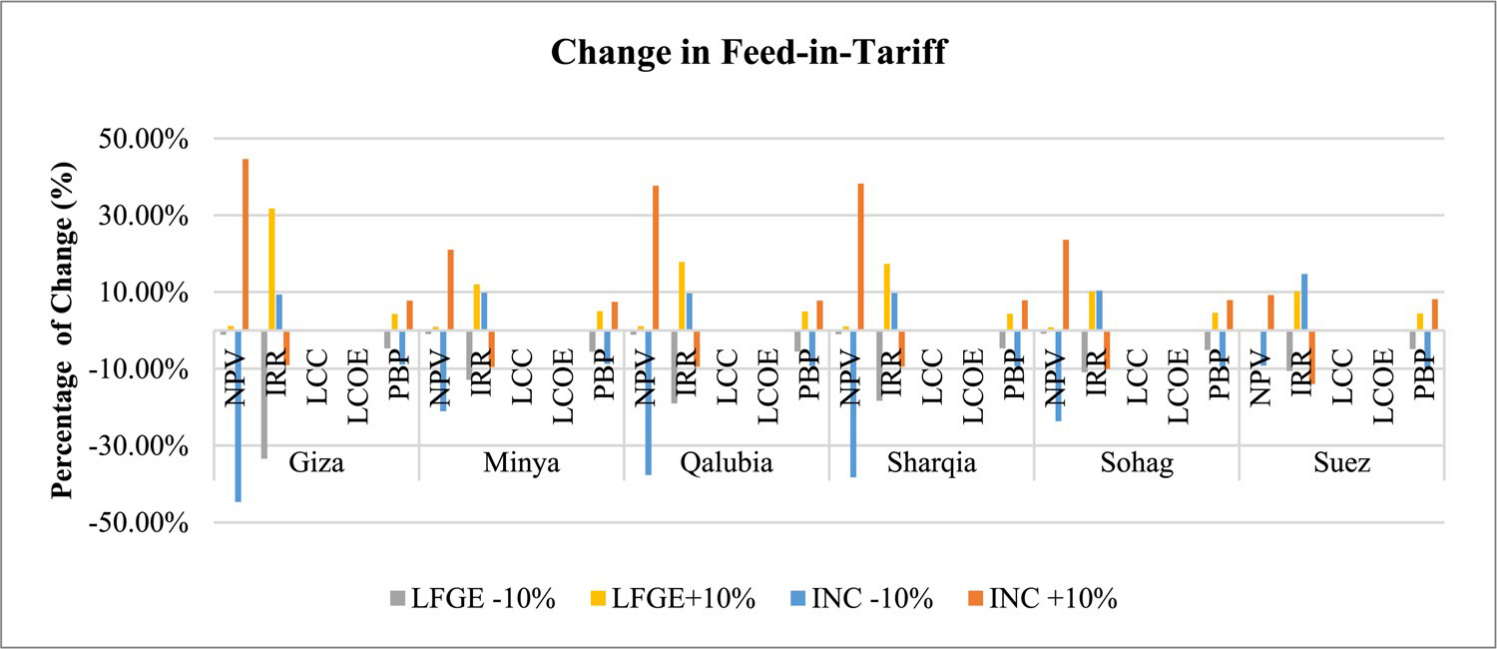
Sensitivity of changing Feed-in-Tariff in WtE profitability.

*FiT Sensitivity*: Fig. 9 indicates that IRR, PBP, and NPV have a varying sensitivity to change in FiT values depending on the WtE technology, with a general trend favoring increased FiT values. For LFGE, IRR is the most sensitive indicator to FiT changes, followed by PBP, and NPV. For instance, a 10% increase in FiT for LFGE would increase the IRR in a range from 10% in Suez to 32% in Giza. In contrast, for incineration technology, NPV is the most sensitive indicator, followed by IRR and PBP. A 10% increase in FiT for incineration projects significantly elevates NPV, ranging from 9% in Suez to 45% in Giza. This sensitivity is due to the direct impact of FiT on projected revenue, with higher FiT values leading to increased revenue for the same amount of energy produced, thereby enhancing project profitability. LCC and LCOE show minimal sensitivity to changes in FiT rates.

**Fig. 10:**
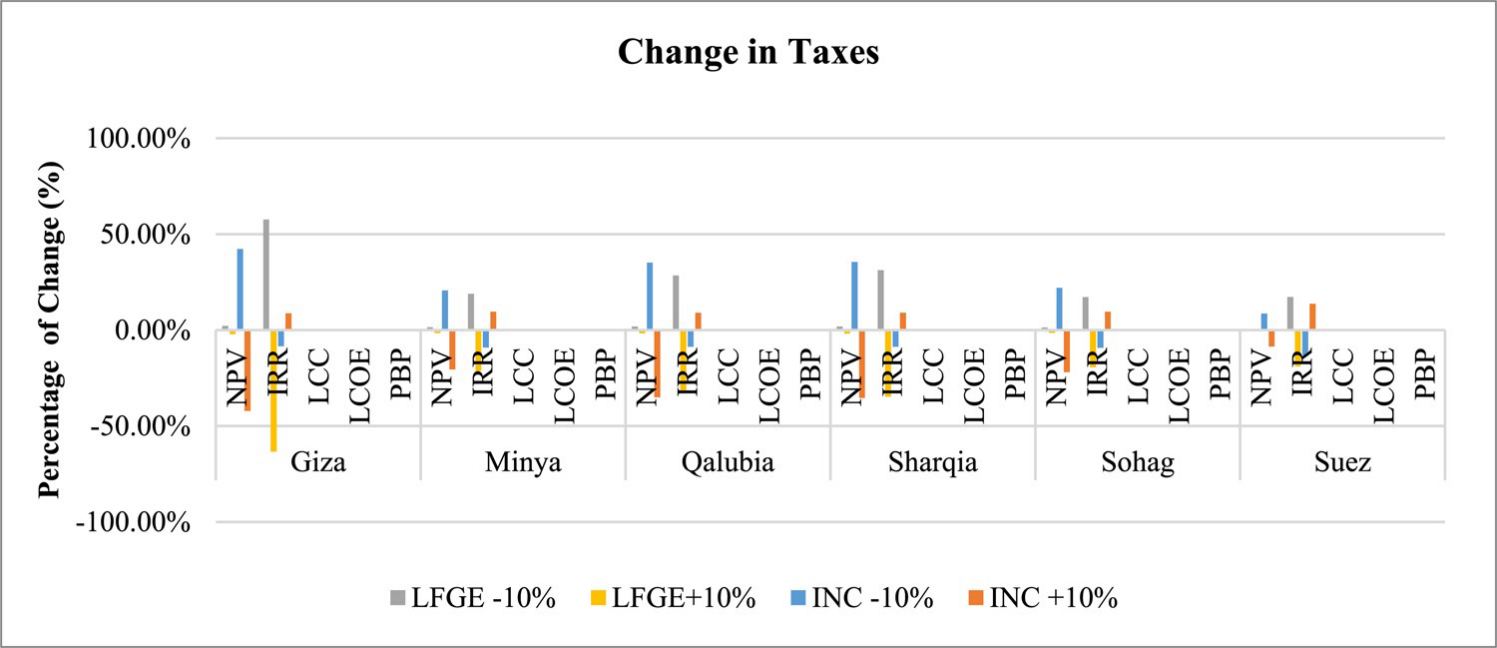
Sensitivity of changing Taxes in WtE profitability.

*Tax Sensitivity*: Fig. 10 depicts the significant impact of tax changes on both NPV and IRR of WtE projects. IRR is more sensitive to tax changes in LFGE projects, with a 10% tax reduction significantly increasing IRR by about 17.1% in Sohag to 57% in Giza. Conversely, NPV is more sensitive to tax changes in incineration projects, with a 10% tax reduction substantially increasing NPV by about 8.5% in Suez to 42% in Giza. LCC, LCOE, and PBP show minimal sensitivity to changes in tax rates.

**Fig. 11:**
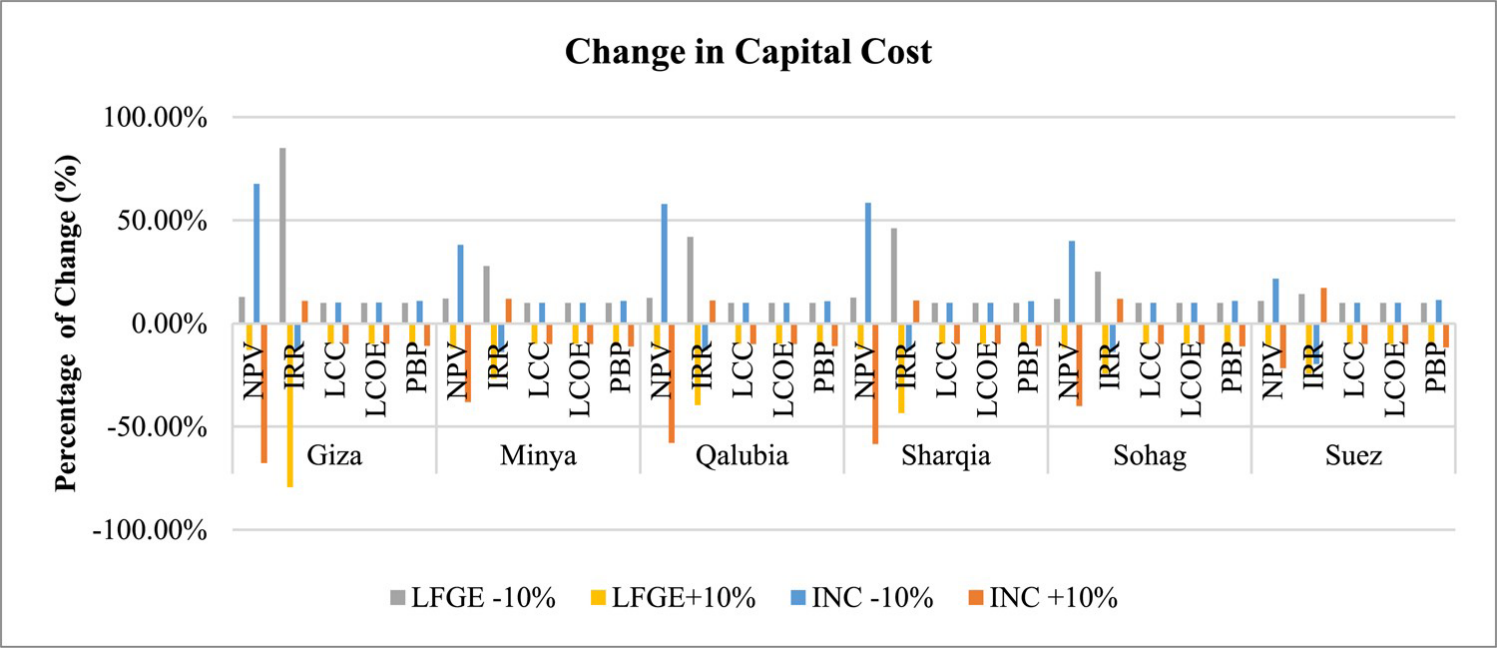
Sensitivity of changing capital cost in WtE profitability.

**Fig. 12:**
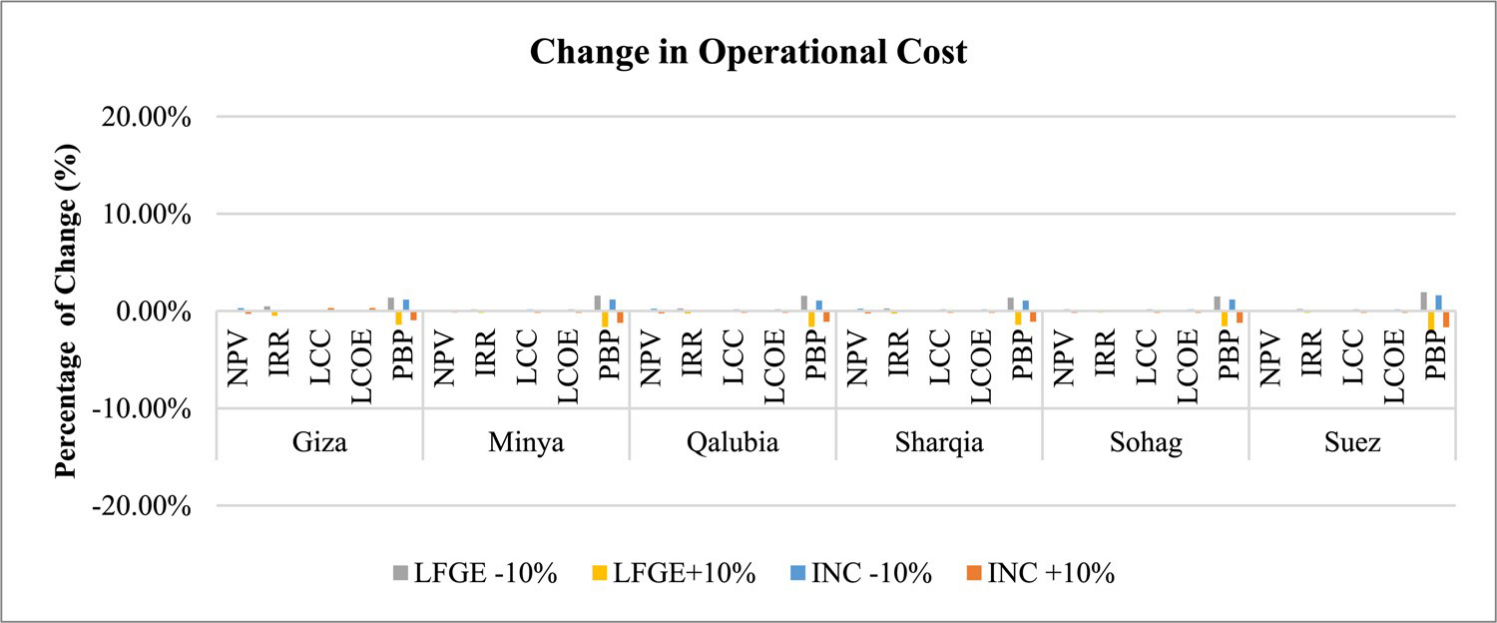
Sensitivity of changing operational cost in WtE profitability.

*Capital and Operational Costs Sensitivity*: Figs. 11 and 12 indicate that economic indicators are more sensitive to changes in capital costs than operational and maintenance costs. In LFGE projects, IRR is the most sensitive indicator to capital cost changes, followed by NPV, with LCC, LCOE, and PBP showing lower sensitivity. A 10% decrease in capital cost could elevate IRR by 14% in Suez to 85% in Giza. For incineration projects, NPV is the most sensitive indicator to capital cost changes, with a 10% reduction in capital cost significantly increasing NPV by about 22% in Suez to 67% in Giza.

*Overall Implications*: Despite the positive changes theoretically observed in NPV, IRR, and PBP across all governorates for the economic variables addressed, most WtE projects still do not achieve a positive NPV or an IRR higher than the discount rate. This outcome is likely due to the substantial initial investment costs associated with WtE projects. Practically, for policymakers and investors, these findings underscore the importance of strategic financial planning, including securing favorable discount rates and FiT values, to improve the financial viability of WtE projects.

### Technical factors

**Fig. 13:**
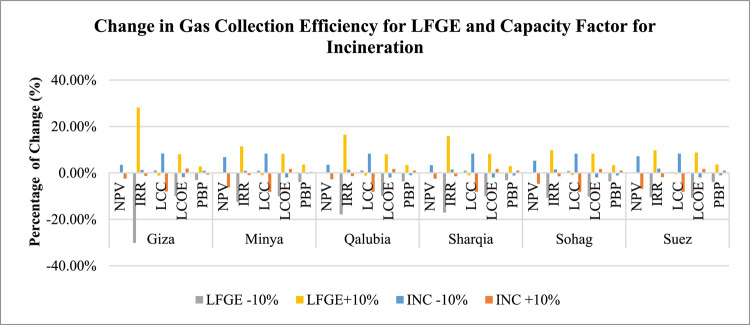
Sensitivity of changing LFGE’s gas collection and incineration’s capacity factor in WtE profitability.

**Fig. 14:**
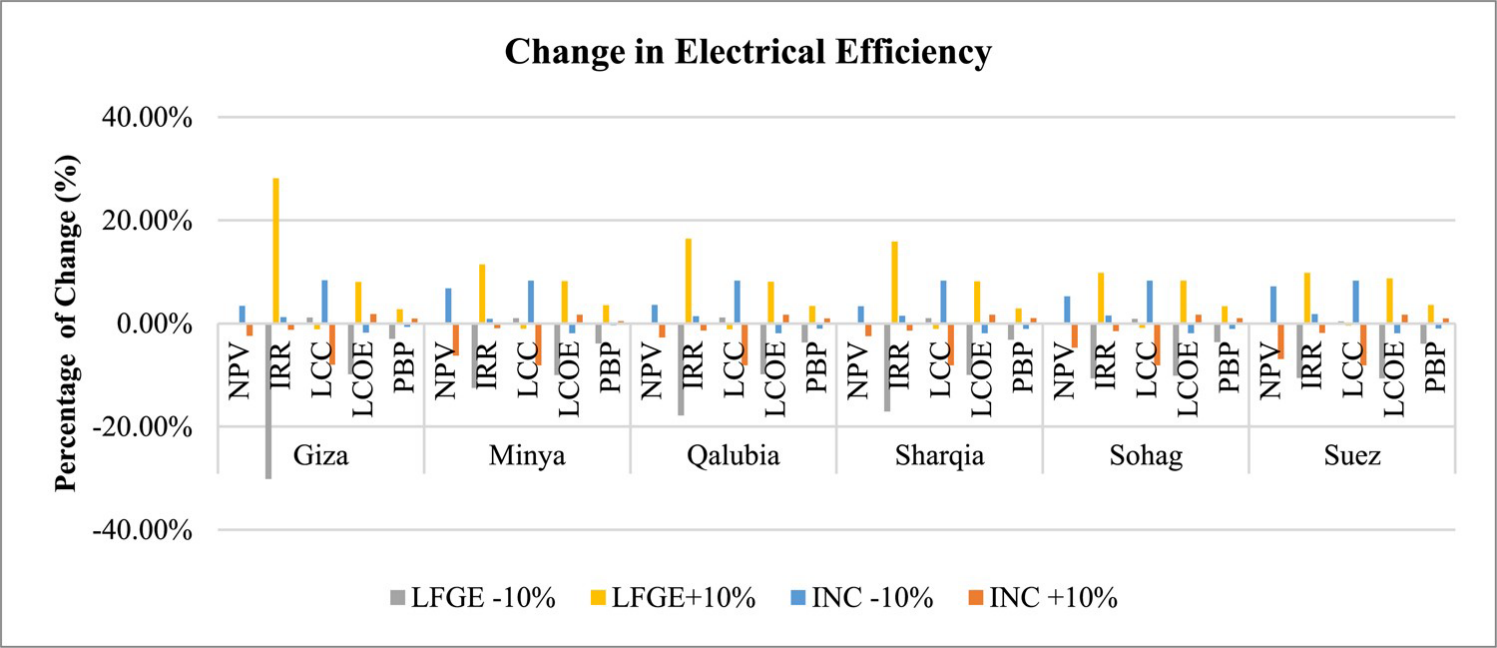
Sensitivity of changing electrical efficiency in WtE profitability.

Regarding the sensitivity analysis conducted for technical aspects, Table 12 presents the outcomes of this analysis including the change in the gas collection efficiency of LFGE, capacity factor of incineration technology, and electrical efficiency of WtE technology. Figures 13 and 14 generally indicates that the change in technical aspects affects similarly in the economic indicators in LFGE and incineration.

**Table 12:** Results of sensitivity analysis conducted for technical factors.

		NPV (M$)		IRR (%)		LCC (M$)		LCOE ($/kWh)		PBP (Y)	
		LFGE	INC	LFGE	INC	LFGE	INC	LFGE	INC	LFGE	INC
		Giza									
Base value		− 393.80	− 120.14	− 1.48%	16.02%	221.55	354.52	1.398	0.241	7.011	2.242
Gas collection efficiency	− 10%	− 393.57		− 1.93%		219.03		1.535		7.221	
	+ 10%	− 394.04		− 1.06%		224.07		1.285		6.818	
Capacity factor	− 10%		− 116.06		15.82%		324.78		0.246		2.221
	+ 10%		− 123.02		16.21%		382.87		0.237		2.257
Electrical efficiency	− 10%	− 393.57	− 116.06	− 1.93%	15.82%	219.03	324.78	1.535	0.246	7.221	2.257
	+ 10%	− 394.04	− 123.02	− 1.06%	16.21%	224.07	382.87	1.285	0.237	6.818	2.221
		Minya									
Base Value		− 330.63	− 94.13	− 5.05%	13.36%	173.75	156.26	1.607	0.288	9.447	2.604
Gas collection efficiency	− 10%	− 330.47		− 5.68%		172.03		1.768		9.813	
	+ 10%	− 330.79		− 4.48%		175.47		1.475		9.113	
Capacity factor	− 10%		− 87.75		13.24%		143.32		0.294		2.613
	+ 10%		− 99.98		13.48%		168.96		0.284		2.592
Electrical efficiency	− 10%	− 330.47	− 87.75	− 5.68%	13.24%	172.03	143.32	1.768	0.294	9.813	2.613
	+ 10%	− 330.79	− 99.98	− 4.48%	13.48%	175.47	168.96	1.475	0.284	9.113	2.592
		Qalubia									
Base value		− 406.79	− 131.14	− 3.12%	15.51%	220.79	331.01	1.401	0.245	8.151	2.320
Gas collection efficiency	− 10%	− 406.52		− 3.68%		218.28		1.539		8.450	
	+ 10%	− 407.05		− 2.61%		223.29		1.288		7.878	
Capacity factor	− 10%		− 126.46		15.29%		303.62		0.249		2.343
	+ 10%		− 134.70		15.72%		357.92		0.240		2.298
Electrical efficiency	− 10%	− 406.52	− 126.46	− 3.68%	15.29%	218.28	303.62	1.539	0.249	8.450	2.343
	+ 10%	− 407.05	− 134.70	− 2.61%	15.72%	223.29	357.92	1.288	0.240	7.878	2.298
		Sharqia									
Base value		− 339.26	− 133.81	− 2.82%	15.55%	185.18	340.66	1.549	0.243	7.830	2.318
Gas collection efficiency	− 10%	− 339.07		− 3.31%		183.28		1.703		8.075	
	+ 10%	− 339.45		− 2.38%		187.08		1.422		7.603	
Capacity factor	− 10%		− 129.37		15.32%		312.47		0.248		2.343
	+ 10%		− 137.09		15.77%		368.35		0.239		2.294
Electrical efficiency	− 10%	− 339.07	− 129.37	− 3.31%	15.32%	183.28	312.47	1.703	0.248	8.075	2.343
	+ 10%	− 339.45	− 137.09	− 2.38%	15.77%	187.08	368.35	1.422	0.239	7.603	2.294
		Sohag									
Base value		− 273.04	− 122.84	− 5.73%	13.73%	141.90	213.90	1.811	0.269	9.852	2.587
Gas collection efficiency	− 10%	− 272.91		− 6.34%		140.66		1.995		10.209	
	+ 10%	− 273.18		− 5.17%		143.15		1.661		9.526	
Capacity factor	− 10%		− 116.38		13.52%		196.20		0.274		2.614
	+ 10%		− 128.58		13.93%		231.29		0.265		2.560
Electrical efficiency	− 10%	− 272.91	− 116.38	− 6.34%	13.52%	140.66	196.20	1.995	0.274	10.209	2.614
	+ 10%	− 273.18	− 128.58	− 5.17%	13.93%	143.15	231.29	1.661	0.265	9.526	2.560
		Suez									
Base value		− 93.16	− 48.86	− 18.00%	8.36%	44.19	46.29	3.862	0.377	20.102	3.733
Gas collection efficiency	− 10%	− 93.14		− 19.90%		44.01		4.273		20.887	
	+ 10%	− 93.18		− 16.24%		44.37		3.525		19.380	
Capacity factor	− 10%		− 45.35		8.21%		42.46		0.384		3.769
	+ 10%		− 52.21		8.51%		50.05		0.370		3.696
Electrical efficiency	− 10%	− 93.14	− 45.35	− 19.90%	8.21%	44.01	42.46	4.273	0.384	20.887	3.769
	+ 10%	− 93.18	− 52.21	− 16.24%	8.51%	44.37	50.05	3.525	0.370	19.380	3.696

*Gas Collection and Electrical Efficiency Sensitivity of LFGE*: IRR and LCOE are sensitive to changes in both gas collection efficiency and electrical efficiency of the LFGE technology, while the rest of economic indicators are not significantly sensitive. A 10% increase in either gas collection or electrical efficiency in LFGE would increase the IRR in the range of 9% in Sohag and Suez to 28% in Giza and increase LCOE by 8% in all governorates.

*Capacity Factor and Electrical Efficiency Sensitivity*: NPV and LCC are sensitive to change in both capacity factor and electrical efficiency of the incineration technology, with both favoring the increase of efficiency, while the rest of economic indicators are not significantly sensitive. A 10% increase in either capacity factor or electrical efficiency would increase the LCC by 8% in all governorates and increase NPV in the range of 3% in Giza, Qalubia, and Sharqia to 7% in Suez.

*Overall Implications*: These results highlight the positive correlation between the increased efficiency in the technical aspects of the WtE technology and improvements in various economic indicators such as NPV, IRR, LCC, and LCOE, which is likely driven by increased energy production revenue. These findings are consistent with similar studies conducted in developing economies, which also report significant impacts of technical efficiency on economic viability^40^. Despite the positive changes theoretically observed in the base values of NPV, IRR, and PBP across all governorates for all technical variables addressed, most projects still do not achieve a positive NPV or an IRR higher than the discount rate. This outcome may be attributed to the substantial initial investment costs associated with WtE projects. Practically, these findings suggest that policymakers and investors should focus on improving technical efficiencies and consider the substantial initial investment costs when planning WtE projects.

#### Policy factors

The sensitivity analysis conducted for the policy aspects is depicted in Table 13 and includes the change in CER certificates and the inclusion of gate fee for WtE technologies.

**Table 13:** Results of sensitivity analysis conducted for policy factors.

		NPV (M$)		IRR (%)		LCC (M$)		LCOE ($/kWh)		PBP (Y)	
		LFGE	INC	LFGE	INC	LFGE	INC	LFGE	INC	LFGE	INC
		Giza									
Base value		− 393.80	− 120.14	− 1.48%	16.02%	221.55	354.52	1.398	0.241	7.011	2.242
Gate fee	50 $/twaste	275.10	548.76	29.53%	32.81%	221.55	354.09	1.398	0.241	1.139	2.240
	100 $/twaste	944.01	1,217.67	54.87%	48.80%	221.55	354.09	1.398	0.241	0.620	2.240
CER price	5 $/tCO2 eq	− 451.33	− 250.66	− 12.40%	12.41%	221.55	354.09	1.398	0.241	16.422	2.867
	99 $/tCO2 eq	− 243.36	221.22	8.26%	23.80%	221.55	354.09	1.398	0.241	2.806	1.424
		Minya									
Base value		− 330.63	− 94.13	− 5.05%	13.36%	173.75	156.26	1.607	0.288	9.447	2.604
Gate fee	50 $/twaste	16.35	252.86	19.95%	33.28%	173.75	156.26	1.607	0.288	1.688	2.604
	100 $/twaste	363.34	599.84	37.15%	52.03%	173.75	156.26	1.607	0.288	0.927	2.604
CER price	5 $/tCO2 eq	− 361.56	− 149.89	− 17.54%	9.53%	173.75	156.26	1.607	0.288	20.070	3.475
	99 $/tCO2 eq	− 249.76	51.70	4.46%	21.11%	173.75	156.26	1.607	0.288	3.962	1.572
		Qalubia									
Base value		− 406.79	− 131.14	− 3.12%	15.51%	220.79	331.01	1.401	0.245	8.151	2.320
Gate fee	50 $/twaste	106.49	382.13	23.23%	29.54%	220.79	331.01	1.401	0.245	1.451	2.320
	100 $/twaste	619.76	895.41	42.93%	42.74%	220.79	331.01	1.401	0.245	0.796	2.320
CER price	5 $/tCO2 eq	− 452.40	− 243.54	− 13.26%	12.11%	220.79	331.01	1.401	0.245	17.360	2.927
	99 $/tCO2 eq	− 287.50	162.83	6.12%	22.93%	220.79	331.01	1.401	0.245	3.414	1.505
		Sharqia									
Base value		− 339.26	− 133.81	− 2.82%	15.55%	185.18	340.66	1.549	0.243	7.830	2.318
Gate fee	50 $/twaste	159.97	365.42	26.49%	28.86%	185.18	340.66	1.549	0.243	1.274	2.318
	100 $/twaste	659.20	864.65	49.21%	41.36%	185.18	340.66	1.549	0.243	0.694	2.318
CER price	5 $/tCO2 eq	− 382.23	− 247.76	− 14.93%	12.20%	185.18	340.66	1.549	0.243	18.297	2.910
	99 $/tCO2 eq	− 226.89	164.21	6.98%	22.91%	185.18	340.66	1.549	0.243	3.137	1.514
		Sohag									
Base value		− 273.04	− 122.84	− 5.73%	13.73%	141.90	213.90	1.811	0.269	9.852	2.587
Gate fee	50 $/twaste	17.37	167.57	20.24%	26.37%	141.90	213.90	1.811	0.269	1.666	2.587
	100 $/twaste	307.78	457.98	37.87%	38.04%	141.90	213.90	1.811	0.269	0.910	2.587
CER price	5 $/tCO2 eq	− 298.39	− 188.00	− 7.00%	10.48%	141.90	213.90	1.811	0.269	22.119	3.261
	99 $/tCO2 eq	− 206.74	47.58	4.22%	20.74%	141.90	213.90	1.811	0.269	4.021	1.679
		Suez									
Base value		− 93.16	− 48.86	− 18.00%	8.36%	44.19	46.29	3.862	0.377	20.102	3.733
Gate fee	50 $/twaste	− 43.38	0.91	9.42%	19.57%	44.19	46.29	3.862	0.377	3.084	3.733
	100 $/twaste	6.39	50.69	20.87%	29.17%	44.19	46.29	3.862	0.377	1.670	3.733
CER price	5 $/tCO2 eq	− 97.42	− 59.31	− 17.00%	5.28%	44.19	46.29	3.862	0.377	51.652	4.799
	99 $/tCO2 eq	− 82.01	− 21.54	− 3.36%	14.60%	44.19	46.29	3.862	0.377	7.739	2.361

**Fig. 15:**
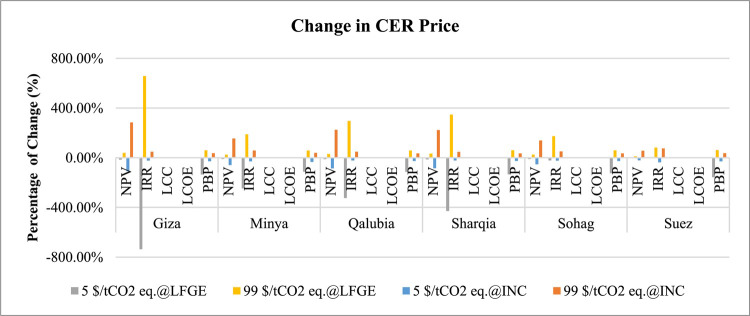
Sensitivity of changing CER certificate price in WtE profitability.

*CER Certificates Sensitivity*: Fig. 15 indicates that an increase in CER certificate prices significantly impacts NPV, IRR, and PBP for both WtE technologies. For LFGE, a high CER certificate price (99 USD/tCO_2_e) significantly increased the IRR, ranging from 81% in Suez to 657% in Giza, and also increased NPV, ranging from 12% in Suez to 38% in Giza. Despite these positive changes, the NPV and IRR values still do not exceed the discount rate, likely due to the substantial initial investment costs in LFGE projects. For incineration, the high CER certificate price substantially increased NPV, ranging from 56% in Suez to 284% in Giza, and increased IRR, ranging from 47% in Qalubia and Sharqia to 74% in Suez. In this case, the NPV turned positive in all governorates, with IRR values exceeding the discount rate and a short payback period, although the LCOE remains higher than the FiT, resulting in a narrow profit margin.

**Fig. 16:**
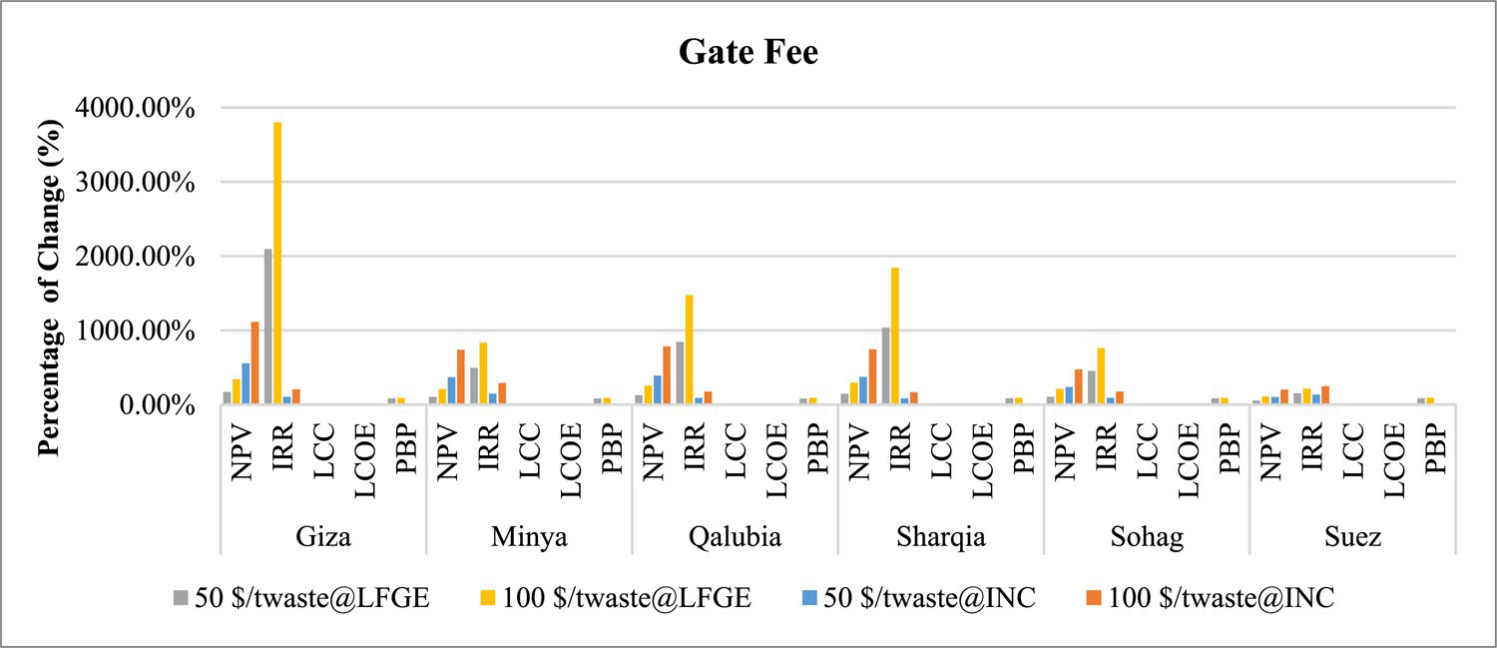
Sensitivity of the inclusion of gate fee in WtE profitability.

*Gate Fee Inclusion*: The inclusion of a gate fee at the examined values showed a major increase in NPV and IRR for both WtE technologies, as depicted in Fig. 16. Similar to the CER certificates, the NPV turned positive for all governorates, with IRR values exceeding the discount rate and a short payback period, although the LCOE remains higher than the FiT. These results suggest that the inclusion of a gate fee could make the proposed WtE projects profitable in the governorates under study.

To further assess this issue, several gate fees were examined to determine the minimum gate fee at which WtE technology could be viable in each governorate, as depicted in Table 14. The table indicates that each governorate has a specific gate fee that makes WtE viable. The minimum gate fee for LFGE projects ranges from 30 USD/tonne of MSW in Giza to 94 USD/tonne in Suez, while the minimum gate fee for incineration projects ranges from 9 USD/tonne of MSW in Giza to 50 USD/tonne in Suez.

**Table 14:** Minimum gate fee for WtE project to be economically viable.

		Min. gate fee ($/twaste)	NPV	IRR	LCC	LCOE	PBP
Giza	LFGE	30	7,540,151	19%	221,546,009	1.40	1.71
	INC	9	259,614	19%	354,088,238	0.24	2.24
Minya	LFGE	48	2,472,802	19%	173,749,781	1.61	1.75
	INC	14	3,027,661	19%	156,255,220	0.29	2.60
Qalubia	LFGE	40	3,833,027	19%	220,786,133	1.40	1.74
	INC	13	2,309,786	19%	331,014,699	0.24	2.32
Sharqia	LFGE	34	214,604	19%	185,183,500	1.55	1.74
	INC	14	5,977,144	19%	340,661,140	0.24	2.32
Sohag	LFGE	48	5,752,815	19%	141,901,052	1.81	1.72
	INC	22	4,939,938	20%	213,902,797	0.27	2.59
Suez	LFGE	94	418,177	20%	44,191,994	3.86	1.77
	INC	50	914,646	20%	46,290,227	0.38	3.73

*FiT Rate Reform*: To address the issue of competitive FiT, decision-makers should consider reforms in the FiT rates, as shown in Table 15. The minimum FiT should range from 1.4 to 3.9 USD/kWh for LFGE projects and from 0.2 to 0.4 USD/kWh for incineration projects to exceed the computed LCOE for both WtE technologies, providing a considerable profit margin for investors.

**Table 15:** Minimum FiT for WtE project to be economically viable.

		Min. FiT ($/kWh)	NPV	IRR	LCC	LCOE	PBP
Giza	LFGE	1.4	197,687,821	21%	221,546,009	1.40	1.02
	INC	0.2	549,004,118	33%	354,088,238	0.24	1.10
Minya	LFGE	1.6	188,098,875	22%	173,749,781	1.61	0.96
	INC	0.3	374,923,090	40%	156,255,220	0.29	0.90
Qalubia	LFGE	1.4	245,696,581	22%	220,786,133	1.40	0.94
	INC	0.2	485,217,313	32%	331,014,699	0.24	1.13
Sharqia	LFGE	1.5	194,808,538	21%	185,183,500	1.55	0.96
	INC	0.2	504,526,709	32%	340,661,140	0.24	1.13
Sohag	LFGE	1.8	149,749,910	21%	141,901,052	1.81	0.96
	INC	0.3	565,081,021	42%	213,902,797	0.27	0.85
Suez	LFGE	3.9	44,953,794	21%	44,191,994	3.86	0.96
	INC	0.4	107,949,948	40%	46,290,227	0.38	0.91

*Overall Implications*: These results theoretically highlight the significant impact of policy adjustments on the economic viability of WtE projects. The inclusion of gate fees and competitive FiT rates are crucial for making these projects financially feasible, which are align with the findings of^36^, which also emphasize the importance of policy support for the economic viability of WtE projects. Practically, policymakers and investors should focus on implementing supportive policies, such as competitive FiT rates and gate fees, to improve the financial viability of WtE projects.

### Achievement of sustainable development goals

Sustainable development refers to the ability of the current generation to meet its needs without compromising the ability of future generations to do the same^8^. Effective management MSW plays a crucial role in achieving sustainable development across three key pillars: environment, economy, and society^31^. In that regard, Egypt has recently formulated its 2030 sustainable development strategy, with proper waste management and the adoption of circular economy principles as central components^36^.

*Circular Economy*: Circular economy is one of the conditions for achieving sustainable development in waste management filed, due to a number of benefits it offers, including reducing, reusing, and recycling of wastes, energy recovery, reducing and avoiding GHG emissions, as well as enhancing economic growth and jobs creation^8,11^.

*Sustainable Development Goals (SDGs)*: The utilization of WtE technologies contributes to achieving several Sustainable Development Goals (SDGs), including affordable and clean energy sources (SDG 7), industry innovation (SDG 9), and sustainable communities and cities (SDG 11), as the used technology would replace other non-renewable energy sources such as typical thermal power plants and would also encourage research and development to increase the efficiency of such technologies as well as ensure the sustainability of living of the nearby communities. Additionally, the utilization of energy produced from WtE would reduce/avoid GHG emissions that are typically emitted from typical power plants producing the same amount of electricity, which would help in achieving the climate action goal (SDG 13)^8,11,14,36,40^.

### Implementation strategies and managerial insights

To ensure the sustainability, scalability, and replicability of WtE projects, several implementation and managerial strategies can be employed. These include regional adaptation, capacity building, public–private partnership, policy advocacy, and monitoring and evaluation, effective management strategies, project planning and risk management, project execution and budget management, compliance and operational efficiency, community engagement and post-closure monitoring.

*Regional Adaptation*: Regional adaptation involves collaborating with waste management authorities, customizing methane estimations, energy potential assessments, and financial analysis methodologies. This customization accounts for local variations such as waste composition and climatic conditions.

*Capacity Building*: Capacity building focuses on training local experts in emissions estimation and energy potential assessment, fostering knowledge transfer through workshops and webinars fostering knowledge transfer and enhancing local expertise.

*Public–Private Partnerships*: Engaging private sector stakeholders through public–private partnerships facilitates joint ventures for WtE projects. These partnerships can provide the necessary financial and technical support to ensure project success.

*Policy Advocacy*: Policy advocacy is crucial for securing supportive policies at national and regional levels. Emphasizing economic and environmental benefits of WtE projects can help garner the necessary policy support.

*Monitoring and Evaluation*: Establishing performance metrics and regularly assessing progress are essential for adapting strategies as needed. Monitoring and evaluation ensure that projects remain on track and achieve their intended outcomes.

*Effective Management Strategies*: WtE technologies, including LFGE and incineration, necessitate effective management strategies throughout the entire project lifecycle^5^. Managers responsible for implementing WtE technologies must engage with various stakeholders, including local communities, government bodies, environmental agencies, and investors, to promptly address concerns.

*Project Planning and Risk Management*: During the project planning phase, risk assessments should be conducted to identify potential risks related to technology, financing, regulatory compliance, and public perception. Mitigation strategies should then be developed to minimize these risks. Additionally, managers must ensure timely acquisition of necessary licenses, permits, and environmental approvals.

*Project Execution and Budget Management*: Throughout project execution, realistic schedules should be developed, considering construction, commissioning, and operational phases. Delays can impact financial viability and community relations. Effective budget management is crucial due to the significant capital investments associated with WtE projects. Adherence to financial plans and efficient resource allocation are essential.

*Compliance and Operational Efficiency*: Throughout the lifetime of the project, managers should stay informed about evolving laws, emissions standards, and waste disposal guidelines to ensure compliance with environmental regulations as well as the application for CER certificates, which can be traded for avoided GHG emissions at WtE facilities, potentially generating income^14^. Also, to ensure the smooth and efficient operation of WtE facilities, managers should focus on hiring, training, and retaining qualified staff. Operational control standards, especially for potentially risky equipment, should be adopted. Regular monitoring of performance parameters (such as energy generation, emissions, and waste quantities) is essential for resource efficiency and optimization^14^.

*Social Aspects and Community Engagement*: Social aspects play a vital role in project continuity. Managers should foster positive relationships with the local community, address their concerns transparently, and educate the public about WtE benefits, emissions control measures, waste reduction, and GHG emission reduction.

*Decommissioning and Post-Closure Monitoring*: At the end of a WtE facility’s lifetime, decommissioning and post-closure monitoring plans should be developed to minimize impact on surrounding communities.

## Conclusions

This study provides a comprehensive assessment of baseline methane emissions, potential energy production, avoided GHG emissions, and the economic feasibility of the two proposed WtE technologies across six governorates in Egypt. The findings offer valuable insights for decision makers aiming to implement sustainable waste management solutions. The following key conclusions drawn from this study, highlighting contributions to the field, implications for policy and practice, and future research directions:

Contributions to the field:

1. *Baseline Methane Emissions and Energy Potential*: The study highlights the significant variations in waste quantities and methane emissions across different governorates, with Giza showing the highest potential due to its large population and waste generation rate.
2. *Energy Production*: Incineration technology demonstrates a substantially higher energy production potential compared to LFGE, making it a more favorable option for large-scale energy generation from MSW.
3. *GHG Emissions Reduction*: The adoption of incineration technology can lead to a significant reduction in GHG emissions, approximately doubling the reduction achieved by LFGE technology. This underscores the environmental benefits of incineration over LFGE.
4. *Economic Feasibility*: While the economic analysis reveals challenges in the viability of WtE projects under current assumptions, incineration shows promise with positive IRR and PBP values. The study emphasizes the need for policy adjustments, such as competitive FiT rates and the inclusion of gate fees, to enhance economic feasibility.

Implications for policy and practice:

1. *Policy Adjustments*: The study identifies specific policy measures, including competitive FiT rates and minimum gate fees specific for each governorate, that are crucial for making WtE projects economically viable. Decision-makers should consider these adjustments to attract investment and ensure the sustainability of WtE projects.
2. *Technical and Economic Sensitivity*: Sensitivity analyses reveal the critical factors affecting the economic performance of WtE technologies. Understanding these sensitivities can help in optimizing project design and financial planning.
3. *Social Considerations*: The study highlights the importance of considering social aspects, such as community acceptance and public perception, in the implementation of WtE projects. Future research should focus on assessing these social dimensions to provide a holistic view of the benefits and challenges associated with WtE technologies.

## Future research directions

1. *Social Impact Assessment*: Future studies should incorporate comprehensive social impact assessments to gauge public views and acceptance of WtE technologies. This will help in addressing potential social barriers and enhancing community engagement.
2. *Long-term Monitoring and Evaluation*: Continuous monitoring and evaluation of WtE projects are essential to assess their long-term sustainability and scalability. Future research should focus on developing robust monitoring frameworks to track project performance over time.
3. *Technological Innovations*: Exploring advancements in WtE technologies and their integration with other renewable energy sources can further enhance the efficiency and sustainability of waste management systems.

In conclusion, this study provides a robust framework for evaluating the feasibility and sustainability of WtE technologies in Egypt. By addressing both technical and policy-related challenges, the findings contribute to the broader goal of achieving sustainable waste management and energy production in Egypt.

## Data Availability

Data sets generated during the current study are available from the authors on reasonable request. The waste generation rates, and waste characteristics data are available from Waste Management Regulatory Authority (WMRA) but restrictions may apply to the availability of these data, which were used with WMRA’s permission for the current study, and so are not publicly available.
